# NuChart: An R Package to Study Gene Spatial Neighbourhoods with Multi-Omics Annotations

**DOI:** 10.1371/journal.pone.0075146

**Published:** 2013-09-19

**Authors:** Ivan Merelli, Pietro Liò, Luciano Milanesi

**Affiliations:** 1 Institute for Biomedical Technologies, National Research Council, Segrate (Milan), Italy; 2 Computer Laboratory, University of Cambridge, Cambridge, United Kingdom; Semmelweis University, Hungary

## Abstract

Long-range chromosomal associations between genomic regions, and their repositioning in the 3D space of the nucleus, are now considered to be key contributors to the regulation of gene expression and important links have been highlighted with other genomic features involved in DNA rearrangements. Recent Chromosome Conformation Capture (3C) measurements performed with high throughput sequencing (Hi-C) and molecular dynamics studies show that there is a large correlation between colocalization and coregulation of genes, but these important researches are hampered by the lack of biologists-friendly analysis and visualisation software. Here, we describe NuChart, an R package that allows the user to annotate and statistically analyse a list of input genes with information relying on Hi-C data, integrating knowledge about genomic features that are involved in the chromosome spatial organization. NuChart works directly with sequenced reads to identify the related Hi-C fragments, with the aim of creating gene-centric neighbourhood graphs on which multi-omics features can be mapped. Predictions about CTCF binding sites, isochores and cryptic Recombination Signal Sequences are provided directly with the package for mapping, although other annotation data in bed format can be used (such as methylation profiles and histone patterns). Gene expression data can be automatically retrieved and processed from the Gene Expression Omnibus and ArrayExpress repositories to highlight the expression profile of genes in the identified neighbourhood. Moreover, statistical inferences about the graph structure and correlations between its topology and multi-omics features can be performed using Exponential-family Random Graph Models. The Hi-C fragment visualisation provided by NuChart allows the comparisons of cells in different conditions, thus providing the possibility of novel biomarkers identification. NuChart is compliant with the Bioconductor standard and it is freely available at ftp://fileserver.itb.cnr.it/nuchart.

## Introduction

The three-dimensional conformation of chromosomes in the nucleus is important for many cellular processes related to gene expression regulation, including DNA accessibility, epigenetics patterns and chromosome translocations [1,2,3].

In recent years, many experimental techniques have been developed to study the nuclear organization at an unprecedented resolution. In particular, the Chromosome Conformation Capture (3C) technology [4,5] and the subsequent genomic variants (Chromosome Conformation Capture on-Chip [6,7] and Chromosome Conformation Capture Carbon Copy [8,9]) are revealing the correlations between genome structures and biological processes inside the cell. The technology relies on the idea that digestion and re-ligation of fixed chromatin in cells allows the determination of DNA contact frequencies and therefore insight into chromosome topology.

The combination of high-throughput sequencing with these techniques, which is generally called Hi-C, allows the characterization of long-range chromosomal interactions genome-wide [10,11,12]. Hi-C gives information about coupled DNA fragments that are cross-linked together due to spatial proximity, providing data about the chromosomal arrangement in the 3D space of the nucleus. If used in combination with chromatin immunoprecipitation, Hi-C can be employed for focusing the analysis on contacts formed by particular proteins, in a technique that is called ChIA-pet [13,14,15,16].

Hi-C is useful to identify active and non-active genome domains, because chromosomal territories fold distinctively, interact hierarchically as independent units, and contain several genes with correlated expression profiles [17,18]. Both Hi-C measurements and molecular dynamics studies are showing a certain degree of colocalization of coregulated genes [19]. Puzzling this colocalization seems to work well for certain families of genes while for other it remains more difficult to achieve. Therefore, it would be important to use all the available multi-omics information to investigate the colocalization of functionally related genes. Both methylations and histone patterns have a large influence in the spatial organization of the genome in the nucleus, with important differences according to the cell type. But there is growing evidence that also CTCF and cohesin proteins act as genome-wide organizers of chromatin architecture and controls the organization of developmentally regulated intra and inter chromosomal contacts [20,21,22]. This is in accord with experimental Hi-C data, because as demonstrated by Botta and collaborators, fragments are enriched of CTCF binding sites [23]. Chromosomal organization and CTCF distribution are also linked to cancer [24] and nuclear morphology studies of tumour cells are achieving a lot of interest [25].

The 3D information is relevant also for the generation of the immunological diversity, which is possible through the V(D)J recombination mechanism that assembles gene segments into functional immunoglobulin (Ig) and T-cell receptor (TCR) genes. This rearrangement is directed by Recombination Signal Sequences (RSSs), which flank each of the hundreds of potential donor gene segments. DNA repair activities then re-join the breaks at two distant cuts to generate functional genes through chromosomal rearrangements [26]. Errors in V(D)J recombination, including cleavage of cryptic RSSs outside the immunoglobulin and T cell receptor loci, are associated with oncogenic translocations observed in some lymphoid malignancies [27].

Also isochores, large regions of DNA (greater than 300 Kb) with high uniformity in guanine (G) and cytosine (C) content, are probably associated to chromosomal rearrangements because they show a high correlation with DNA breakpoints [28]. Notably, it has been shown that the GC-content of isochores is correlated with many other genomic features: gene density, replication timing, recombinations, methylation patterns, and distribution of transposable elements. Thus, interpreting the mechanism underlying the evolution and spatial organization of isochores is a major issue in understanding the organization of genomes [29].

In the last 12 months few software (see Table 1 for a list and brief description) have appeared that process Hi-C data for showing inter and intra chromosomal interactions with the possibility of loading annotations by employing data in bed file format [30,31,32,33]. Although some studies about the analysis of long-range interaction networks have been presented [34,35], current approaches to Hi-C data analysis mostly rely on the conversion of information into contact maps, which are matrices of pair wise contact frequencies along the genome. Also data normalization is performed directly on the contact maps, with the aim of filtering out biases caused by fragment length, mappability, and GC-content. Here, we present the R package NuChart, which provides a systems biology view of these data with the aim of giving a gene-centric Nuclear Chart of the genome spatial organization. This is firstly accomplished by providing a modified method of normalization, which is adaptive and works directly on Hi-C fragments. Working at sequence level allows exploiting a systemic view to this kind of data, because entire pathways can be mapped into nuclear maps to identify clusters of functionally aggregated genes.

**Table 1:** List of the available software for the analysis of Hi-C data.

Software	Institution	Year	Reference
Hicup	Babraham Bioinformatics	2012	30
HClib	Massachusetts Institute of Technology	2012	31
Homer	University California, San Diego	2012	32
HiCT	Institute Curie	2012	33

NuChart integrates Hi-C information, describing the chromosomal neighbourhood, with predicted CTCF binding sites, isochores, potential cryptic RSSs, and other user-provided genomic features, such as methylation patterns or DNase hypersensitive sites, to infer how the nuclear three-dimensional organization works in controlling gene expression. Moreover, by exploiting the Exponential-family Random Graph Models (ERGMs), NuChart analyses the structure of a neighbourhood graph and also the relation of its topology with respect to the mapped multi-omics features.

A typical question the software attempts to answer is the following: what are the most important genomic features in the space nearby a gene of interest? Given a list of genes or a specific pathway, we can identify their neighbourhood in the 3D nucleus organization by following Hi-C fragments and then identifying the chromosomal domains that relate to the input. This software could also provide information on spatial proximity of gene promoters and the density of highly expressed nearby genes that may point to some important questions such the co-proximity of genes coding for protein interacting pairs with respect to the nuclear pore localisation.

## Design and Implementation

The NuChart package has been designed to provide a novel gene-centric, pathway-oriented, multi-omics tool for the representation and the statistical analysis of Hi-C data. The package contains four sets of functions: (i) the first group to load and normalize data; (ii) the second to create neighbourhood graphs; (iii) the third to map genomic features and expression data on graphs; (iv) and the last one to compare and statistically analyse graphs of different cells or experimental conditions.

The main function to import data into the R environment is *load_HITCSAM_file*, which loads data in the Sequence Alignment Map (SAM) format as provided by the Hicup [30] software. Hicup is a well-established read-based software for Hi-C data pre-analysis, which takes in input the bare FASTA Quality (FASTQ) files and performs the mapping and a preliminary filtering of the sequences. In particular, for each read representing a digested fragment, Hicup analyses the distance from the nearest restriction site to verify if the distance is reliable (it should be less than a fixed threshold, otherwise the corresponding Hi-C contact is filtered out). At the end, each line of the input SAM file contains the information concerning a sequenced read, representing a digested fragment, coupled with its paired by the Hi-C experiment. The *load_HITCSAM_file* function creates a data frame, the main data structure of the NuChart package, on which all the other package functions rely. By employing the *convert_HITCSAM_matrix* function, this data structure can be turned at any time into the corresponding contact map, in order to be analysed with other standard software.

From the normalization point of view, NuChart presents a modified version of the Hu et al. [36] approach (a simplification of the original method of Yaffe and Tanay [37]). This is a parametric model relying on a Poisson statistics and the NuChart normalization works on the same basis, but providing a score to each read, identifying half of the Hi-C contact, instead of normalizing the contact map. This approach allows preserving the sequence information for the creation of the neighbourhood graph and for mapping the genomic features in the following. The rationale is to safeguard through the normalization the information about the sequences instead of blurring the data of the reads within the contact map. Moreover, this scoring approach involves the use of a user-selected threshold, which allows performing a fine tuning of the normalization, verifying which reads are filtered at different values and regulating the algorithm consequently. As for the approach of Hu et al. [36], the NuChart normalization relies on the computation of local genomic features that describe the fragment length, the GC-content and the sequence mappability. At the same way, a normalizing window must be specified, in order to build a local statistics for the parametric model. In order to allow the combination of the two normalization approaches, the Poisson model provided by Hu et al. [36] has been re-implemented in the function *normalize*_*CONTACT*_*map*.

The core of NuChart is the *graph_interaction_genes* function that creates the neighbourhood graph of the genes provided as input. This function creates a graph in which the vertices are the genes and the edges are the Hi-C contacts represented by the reads. Beside the input genes and the sequencing results, the user specifies the restriction enzymes used for the experiment and the related digested fragments (data about the most common enzymes are provided with the package, but for other particular combinations Hicup provides a perl script for computing such files). These data are used to identify which fragments belong to the input genes. Then, using the association matrix represented by the coupled reads, each fragment is associated with another fragment in a different genomic region. If the identified fragment is within a gene an edge is created on the graph between the starting gene and the novel detected one. If the identified fragment is intergenic, the corresponding genomic position is represented on the graph as a singularity point (red dot) that, if the user desires, can be then connected with the nearest upstream and downstream genes in terms of genomic coordinates, which can be very important for the subsequent analysis of colocalization and coregulation.

**Figure 1:**
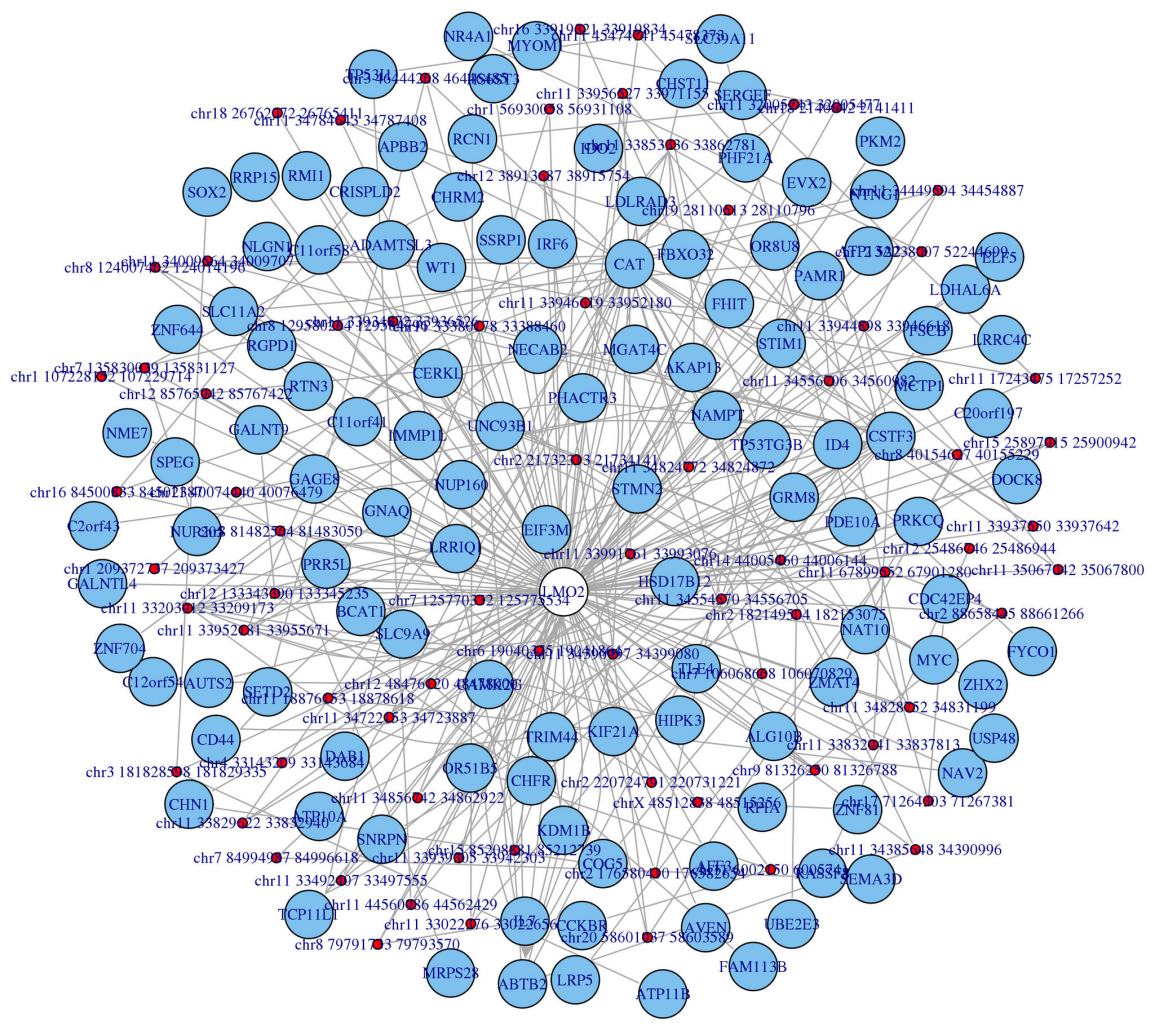
Neighbourhood graph of the gene LMO2 according to the Lieberman-Aiden et al. Hi-C experiment.

In other words, starting from the genomic coordinates of fragments belonging to the input genes, and using experimental associated reads as a cross-reference table, Hi-C connected genes are mapped on the graph. In order to compare genomic coordinates of reads, fragments, and genes, the function *graph_interaction_genes* makes an extensive use of the package biomaRt (although human and mouse data are shipped with the package in order to improve performance). Using this approach it is possible to identify the neighbourhood graph of the genes provided by the user, according to the Hi-C experiment under analysis, overcoming the problem of intergenic contacts by expanding these singularity points to the nearby genes. As an example, Figure 1 shows the neighbourhood of the gene LMO2 according to Lieberman-Aiden et al. [11] Hi-C data (SRA:SRR027963)

As mentioned, the connections established by fragments are mapped as edges in the output graph, while vertices represent the genes connected by fragments. This procedure is performed for all the input genes and can be automatically repeated many times, making the output of one neighbourhood analysis the input for the following iteration. The possibility of iterating this analysis permit to follow progressively the connections provided by Hi-C data, enabling the opportunity to explore increasingly the chromosomal territories that concern the initial set of genes. Noteworthy, the *graph_interaction_genes* function is implemented to take into account genome-wide data, which allows searching both for inter and intra chromosomal fragments. Moreover, the function can deal with multiple connections between genes and also with loops inside single genes, in order to provide a complete genome-wide description of the chromosomal three-dimensional conformations.

In other words, the *graph_interaction_genes* function is able to generate, relying on the *igraph* package, the neighbourhood graph by iterating the Hi-C contact research process according to a user-selected parameters, which allows to extend the analysis far away from the input genes or to focus the attention only to the near proximity of them. The neighbourhood graph can be drawn using the common *plot* function, which can redirect static graphs to any R device, or using *tkplot*, that enables a user-interactive graph drawing facility. Although *igraph* contains a lot of functions and many different options to represent graphs, if the user would like to use a different tool for visualization (such as the R packages *Rgraphviz*, *graph*, and *network* or also *Cytoscape*) the graph can be exported in many formats, by employing the function *write. graph*. Moreover, beside the graph, NuChart provides as output two tables describing respectively the vertices (genes) and the edges (Hi-C contacts) of the neighbourhood graph.

Based on this core implementation, NuChart provides two important functions. The first, *graph_interaction_pathways*, enables the creation of neighbourhood graphs for full-annotated networks, thanks to the possibility of querying both KEGG (using KEGGREST) and REACTOME (using biomaRt) to download the list of genes of a user-selected pathway. Considering the importance that Hi-C profiles can have in describing the spatial conformation of a specific genomic region, NuChart implements also a function for mapping the neighbourhood of specific chromosomal regions. In detail, the function *graph_interaction_coord* allows to select a specific genomic interval in order to create a graph of the Hi-C contacts that involve genes in that region. This feature can be particularly interesting to highlight differences in the organization of chromosomes from a cytogenetic point of view, potentially providing the possibility of identifying novel biomarkers related to the spatial conformations of specific genomic regions.

Due to the large number of operations the *graph_interaction_genes* function must perform for creating the neighbourhood graph, in particular while working with a large number of genes or considering a whole pathway, the main computational routine has been implemented to exploit, if present, the *multicore* package. In detail, there is a switch controlling the entrance in the iterative search for Hi-C contacts in the neighbourhood of a selected genes: if the *multicore* package is loaded into the R environment the function exploits all the available cores for parallelizing the procedure, otherwise the common sequential approach is performed.

Considering the importance that chromosomal territories assume in gene expression coregulation, NuChart provides the possibility to map on the neighbourhood graph expression data from ArrayExpress and Gene Expression Omnibus (GEO). Using the *get_expression* function, it is possible to download expression data from these repositories (using respectively the R/Bioconductor packages ArrayExpress and GEOquery), to perform a standard normalization and a differential expression analysis of them (using *limma* or *samr*), in order to identify down-regulated and up-regulated genes in the neighbourhood. These data are used to provide different weights (according to the logarithm of the fold change) and colours to the vertices of the graph (respectively green is used for down-regulation and red for up-regulation).

**Figure 2:**
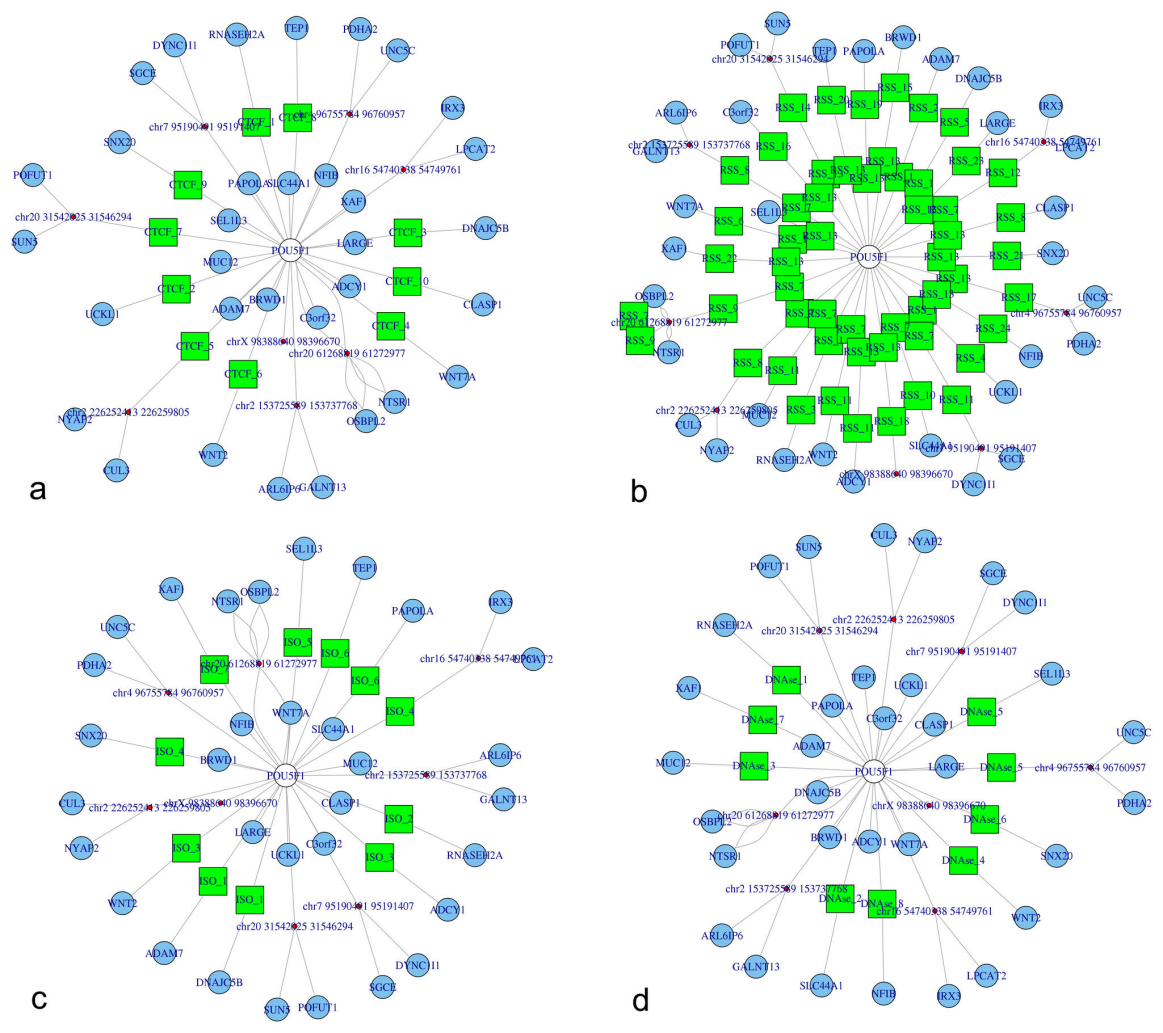
Representation of the OCT4 (official name POU5F1) neighbourhood graphs according to the Dixon et al. experiments with multi-omics annotations. In particular, panel a) represents CTCF binding sites mapping, panel b) cryptic RSSs mapping, panel c) isochores mapping, and panel d) DNase hypersensitive sites mapping.

NuChart also provides the possibility of mapping on the edges of the neighbourhood graph genomic features that are known to be involved in chromosomal recombination, looping and stability. Noteworthy, the software contains the *map_FEATURES_file* function that can be used to load any kind of annotations provided as bed file to characterize the neighbourhood graph. In particular, the package comes with data concerning predicted cryptic RSSs [38], indication of possible CTCF binding sites [39], and isochore distribution [40]. All these genomic features have been pre-computed both on the human (hg19) and murine (mm9) genomes, and are available as bed files, ready to be mapped on the edges of the neighbourhood graphs. For example, Figure 2 shows the OCT4 (official name POU5F1) graph according to Dixon et al. [12] Hi-C data (SRA:SRR400261) annotated with information about CTCF binding sites (a), predicted cryptic RSSs (b), isochores (c) and DNase hypersensitive sites (d). Nonetheless, it is possible to compute on the fly, working directly on the fragment sequences related to each edge, predictions about the presence of cryptic RSSs (*map_RSS_computed*), CTCF binding sites (*map_CTCF_computed*), and enriched GC-contents (*map_ISO_computed*). In order to exploit the computation on the fly of these features, the user must load the BSgenome package concerning the genome under analysis. This is essential while working on species different from human and mouse, but it can be also exploited, by forging a user-defined specific BSgenome data package, to analyse genomes rich of genomic variations, which can have altered genomic features with respect to the normal ones.

The last group of functions has been designed to describe, compare and statistically analyse graphs. The first function *graph_statistics* computes global indexes such as density, connectivity and diameter which describe the graph as a whole, and local indexes, such as node degree, betweenness and closeness that describe in detail the neighbourhood of each gene of the graphs. The second function, *graph_correlation* has been implemented to correlate graph, by transforming them into adjacency matrices and then calculating their Pearson correlation. This is a key aspect because it allows the comparison of the spatial conformation of a gene neighbourhood between different sequencing runs performed on the same cells, between the same cells under different conditions, or between different types of cells. A third function, *graph_ergm*, has been designed to statistically analyse the structure of the neighbourhood graph and the relation between its topology and the annotated multi-omics features. In particular, this function relies on the package *ergm* that provides an integrated set of tools to fit and analyse networks based on the Exponential-family Random Graph Models. Therefore, NuChart can be used to create statistical models of neighbourhood graphs by implementing maximum likelihood estimators, which are calculated using Markov Chain Monte Carlo (MCMC) [41]. Although ERGMs is the default statistical framework for the analysis of graphs, NuChart can be interfaced with other R packages by exploiting the *igraph* data structure.

## Results and Discussion

**Figure 3:**
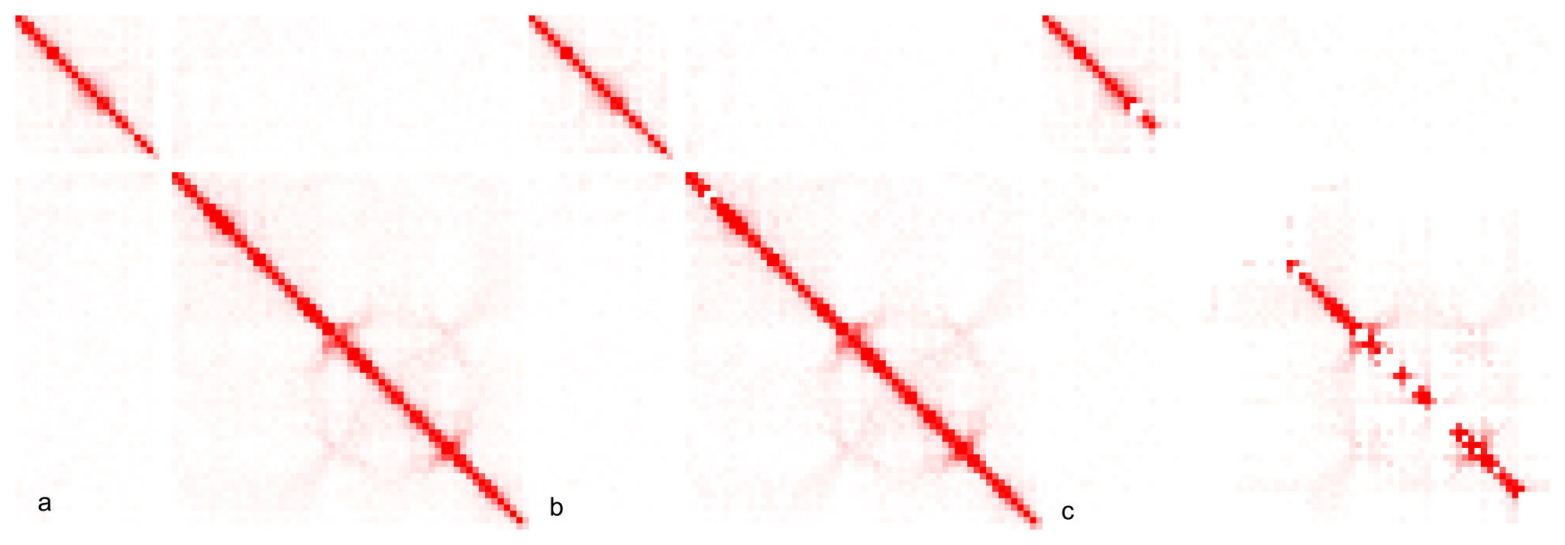
Normalization of chromosome 17 Hi-C data according to the Lieberman-Aiden et al. experiment. In panel a) the Hu et al. normalization is shown, while in panel b) the read-based normalization performed with NuChart (threshold 0.9) is presented to show the reproducibility with respect to the Hu et al. approach. Panel c) represents the NuChart read-based normalization performed using a more restrictive threshold (threshold 0.99).

NuChart has been designed to improve both analysis and representation of Hi-C data, by employing a gene-centric, pathway-oriented approach to the treatment of chromosome capture information A first task addressed by the package is the normalization of data, which still represents an open issue in Hi-C data analysis. We propose a combination of methods that enable an unmet flexibility for the biologist, which can both perform a read-based normalization, selecting the most suitable threshold according to his experience and sensibility, and convert data into a contact maps to combine, if desired, also a matrix based normalization. An example of the reproducibility of the Hu et al. [36] approach for normalization with respect to our read-based approach is presented in Figure 3, which concerns Lieberman-Aiden et al. [11] Hi-C data (SRA:SRR027963) of chromosome 17. In the left panel, the common contact map normalization is presented, in the central one the read-based normalization with a threshold reproducing the Hu et al. [36] approach is shown, while in the right panel a more strict normalization is presented.

**Figure 4:**
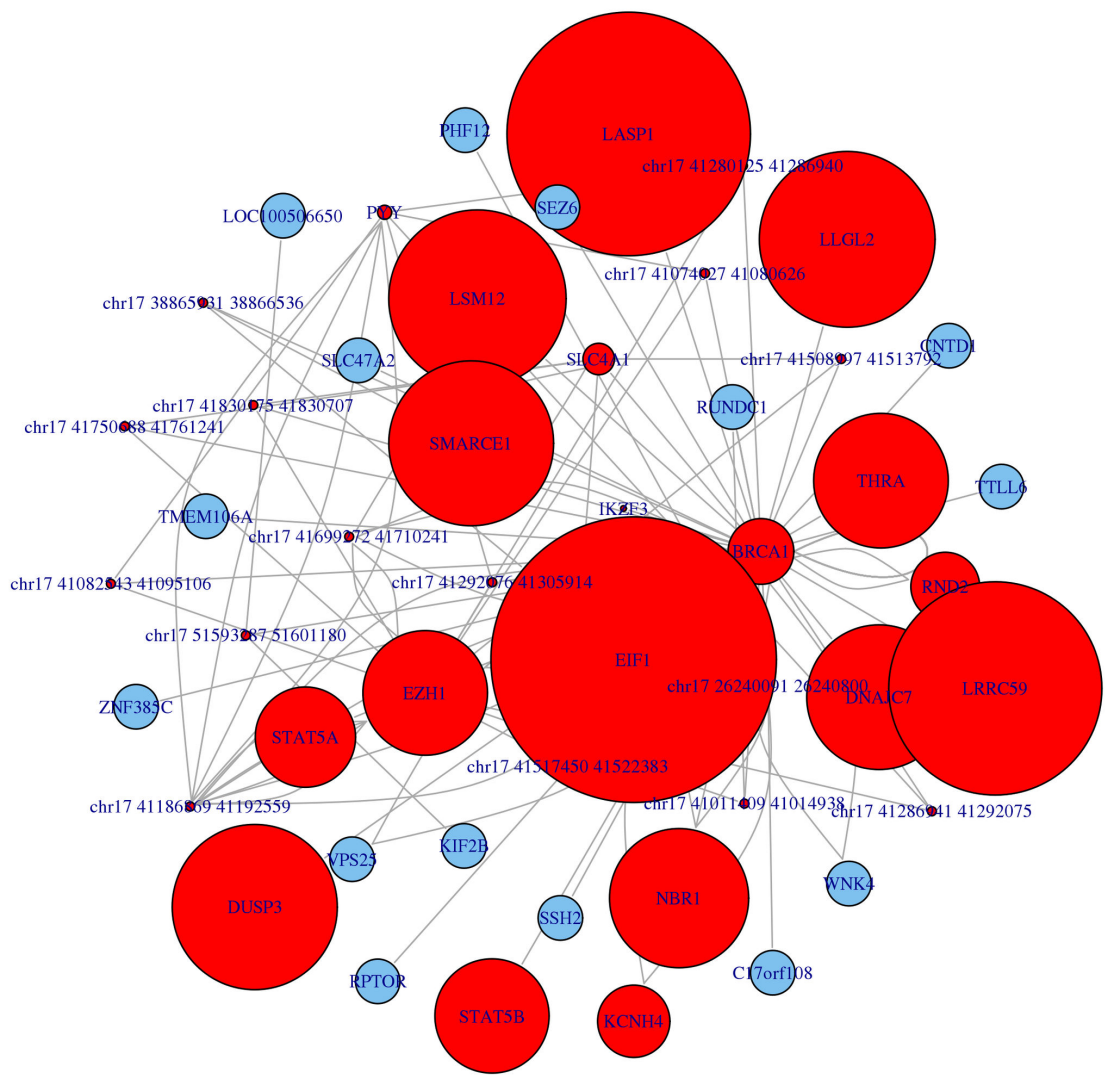
Neighbourhood graph of the gene BRCA1 according to the Lieberman-Aiden et al. experiment. Gene expression data about colon cancer experiment GDS3160 have been mapped on the graph to show the enhanced description (and prediction) power that the graph representation has in relation to gene co-expression with respect to the approach relying on genomic coordinates.

The representation of Hi-C data using a graph approach, oriented at overcoming the common view relying on genomic coordinates, is a step forward the available representation tools, in particular considering the capability of mapping multi-omics features on the graph. The possibility of representing expression profiles on the graph allows analysing the co-expression of mapped genes, providing a strongest correlation than the one provided by the mere chromosomal coordinates. As an example, we show in Figure 4 the neighbourhood graph of the gene BRCA1 according to Lieberman-Aiden et al. [5] Hi-C data (SRA:SRR027963), with mapped the expression profile of the GEO Omnibus experiment GDS3160 related to colon cancer [42].

**Figure 5:**
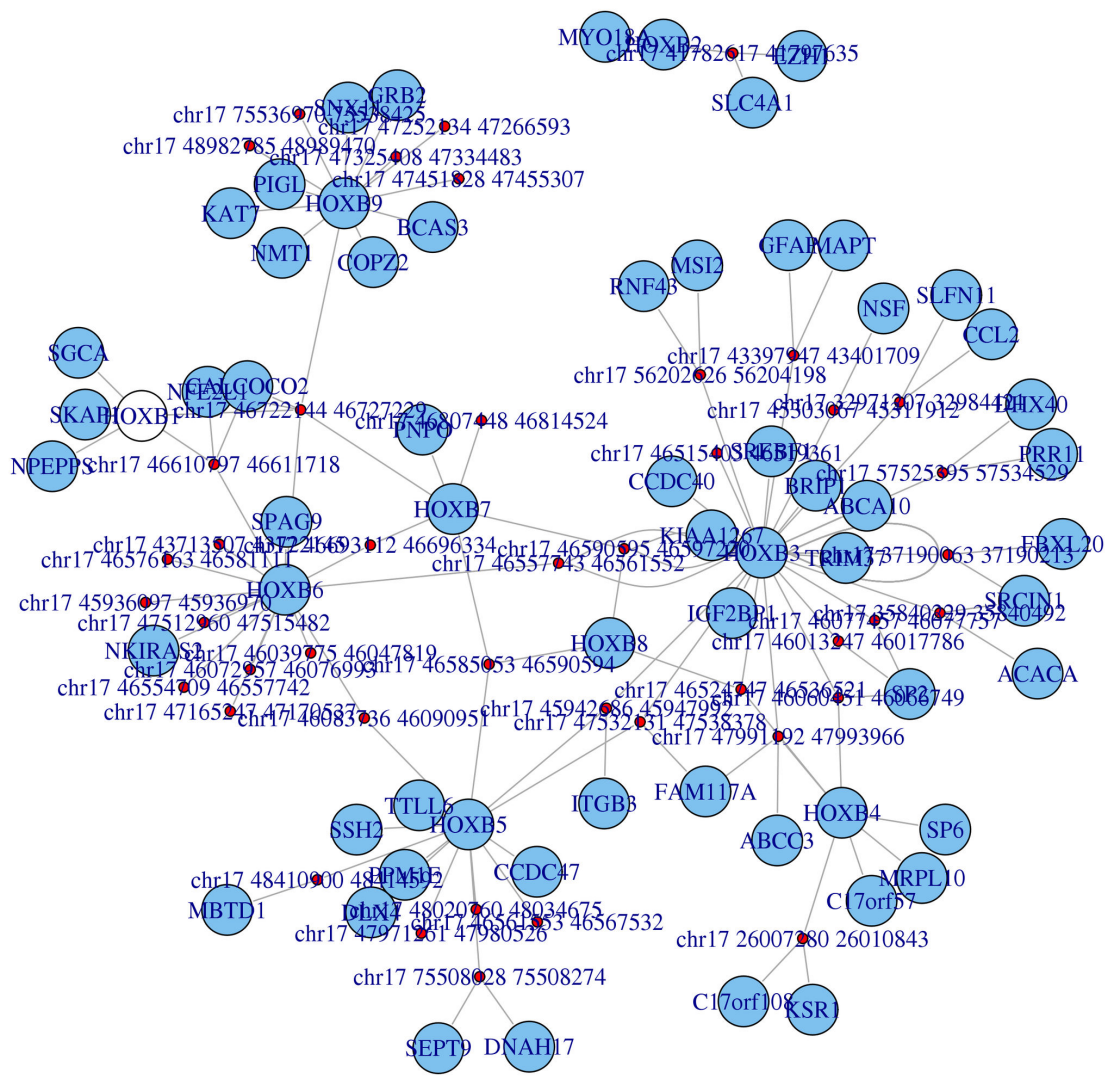
Neighbourhood graph of the 17q21.32 cytoband concerning the Homeobox B cluster (HOXB) of genes according to the Lieberman-Aiden et al. Hi-C experiment.

NuChart can be also very useful for the description of the DNA organization while looking at full chromosomal regions or cytobands. The idea of correlating different states, for example physiological and pathological conditions, is in hand by employing the Hi-C technology. The option of creating a map for a specific cytoband is very innovative and allows the discernment of different cell states at cytological level, potentially providing to researcher novel powerful biomarkers. In Figure 5, as an example, the neighbourhood graph of the Homeobox B cluster (HOXB) of genes (cytoband 17q21.32) is shown according to Lieberman-Aiden et al. [11] Hi-C data (SRA:SRR027963).

**Figure 6:**
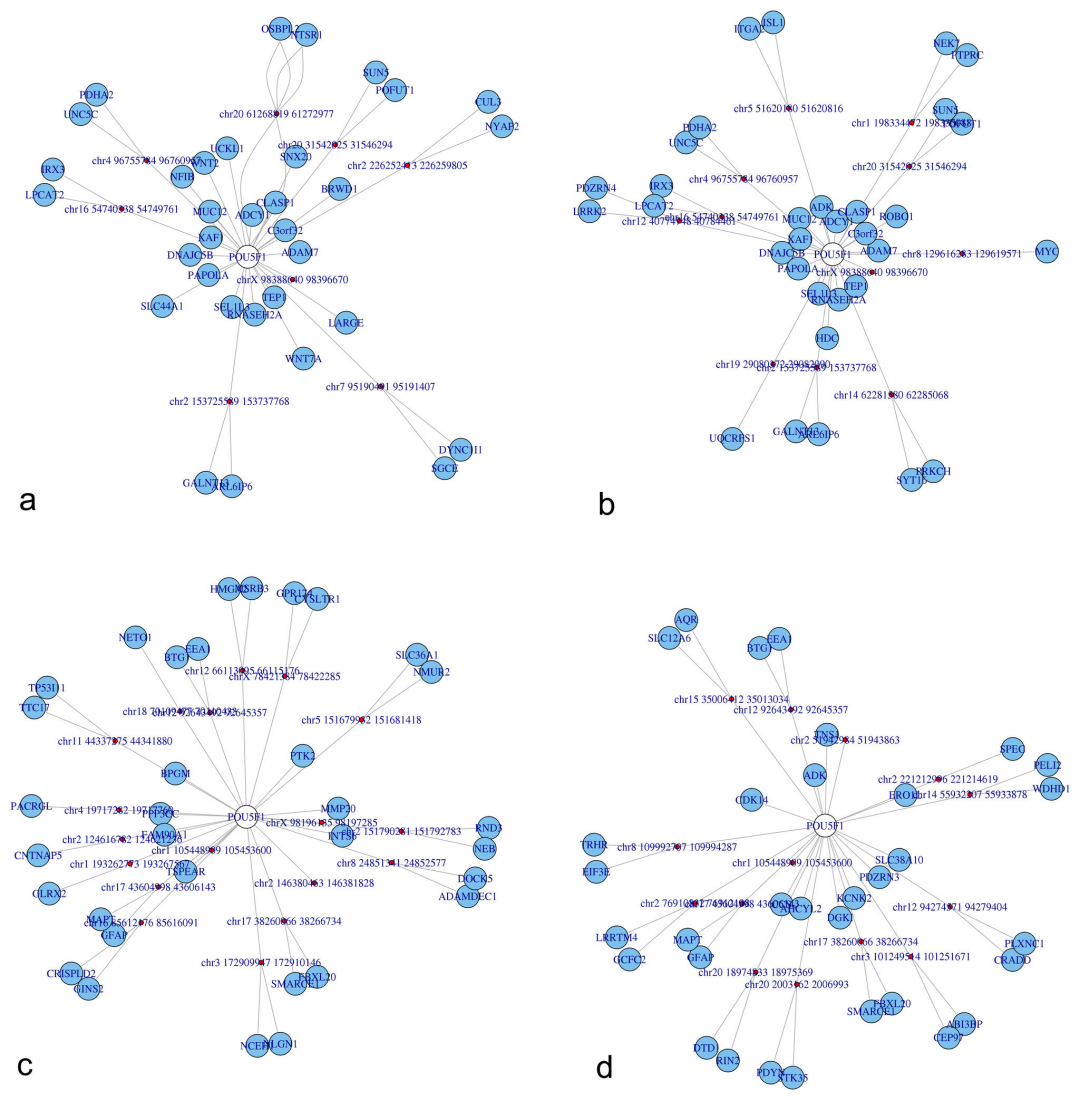
Representation of the OCT4 (official name POU5F1) neighbourhood graphs in four different runs from the Hi-C experiments of Dixon et al. to show inter and intra run modifications. In the panel a) and b) on the top part of the figure, the sequencing runs are from human embryonic stem cells (hESC), while panel c) and d) are from human foetal lung fibroblasts (IMR-90).

The possibility of describing graphs through statistics and, even more, the capability of correlating them through the adjacency matrix representation is a powerful tool to highlight similarities and differences in different Hi-C runs, in different cell conditions or in different cell types. In Figure 6, an example of different graphs for the gene OCT4 achieved according to four different runs of the Dixon et al. [12] experiments is shown. Respectively, the graphs in the top part are from two different runs performed on human embryonic stem cells (hESC; SRA:SRR400261 and SRA:SRR400262), while the graphs in the bottom part are from human foetal lung fibroblasts (IMR90; SRA:SRR400264 and SRA:SRR400265). It is very interesting to see how the gene neighbourhood changes in these four datasets because, as data in Table 2 confirms, there is a considerable similarities between runs, but a substantial uncorrelation between data of the two cell lines.

**Table 2:** Correlations between the neighbourhood graphs of three different genes (OCT4, TP53 and POLR2A) in four different runs of the Dixon et al. experiments (SRR400261, SRR400262 about human embryonic stem cells (hESC) and SRR400263, SRR400264 about human foetal lung fibroblasts (IMR-90)).

OCT4	SRR400261	SRR400262	SRR400264	SRR400265
SRR400261	100%	66.9%	0.5%	0.6%
SRR400262	66.9%	100%	0.4%	7.9%
SRR400264	0.5%	0.4%	100%	40,4%
SRR400265	0.6%	7.9%	40,4%	100%
TP53	SRR400261	SRR400262	SRR400264	SRR400265
SRR400261	100%	33.8%	0.4%	0.5%
SRR400262	33.8%	100%	0.6%	0.7%
SRR400264	0.4%	0.5%	100%	20,4%
SRR400265	0.6%	0.7%	20,4%	100%
POLR2A	SRR400261	SRR400262	SRR400264	SRR400265
SRR400261	100%	50.9%	2.2%	0.2%
SRR400262	50.9%	100%	0.2%	0.2%
SRR400264	2.2%	0.2%	100%	70,4%
SRR400265	0.2%	0.2%	70,4%	100%

Intra runs variability is much lower than inter run variability.

**Figure 7:**
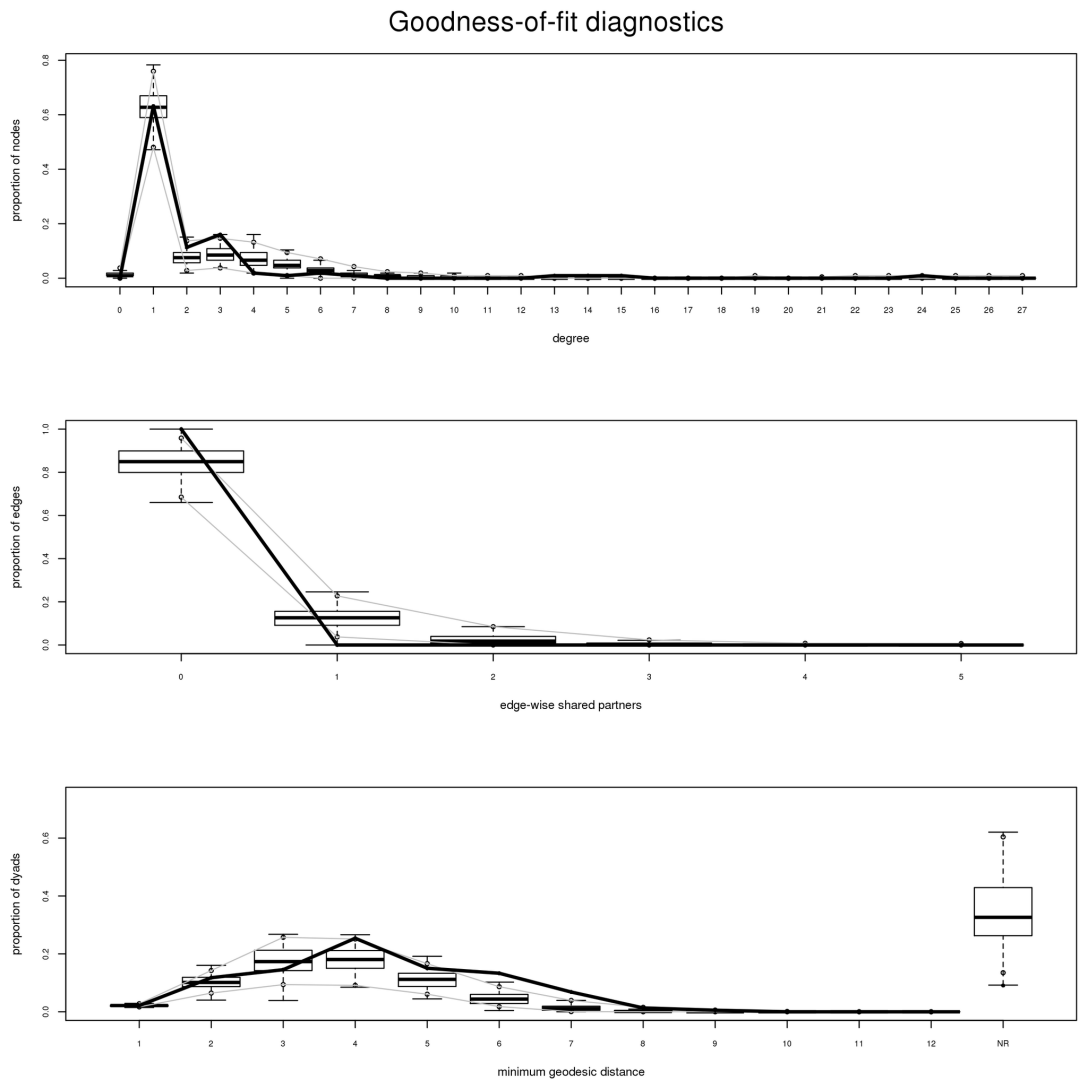
Goodness of fit diagnostics charts for three topological features of the stochastic estimator for the HOXB cluster of genes neighbourhood graph according to the Lieberman-Aiden et al. Hi-C experiment. The thick black line represents the real data concerning the analysed graph, while the boxplot shows the statistical properties of the estimator achieved by employing stochastic simulations. In the top panel the analysis of the estimated model in relation to the degree distribution of the HOXB neighbourhood graph; in the central panel the analysis of the estimated model in relation to weighted edge-wise shared partner statistic; in the bottom panel the analysis of the estimated model in relation to the minimum geodetic distance of the HOXB neighbourhood graph.

By employing ERGMs, NuChart allows the option of statistically analyse the structure of the neighbourhood graph, implementing a stochastic model of the network and using MCMC to create an estimator trough a likelihood function. These models can be used to compute simple statistics about the significance of some graph characteristics, such as the topology of the edges (edges), the vertex tendency to be reciprocal (mutual), the distribution of the vertex degree in the graph (degree), or the measure of vertex clustering attitude (triangle). On the other hand, by choosing more complex modelling functions and exploiting the mapped multi-omics features, the user can test the probabilities that edges are function of a specific genomic feature (nodecov) or the significance of having edges in relation to a particular vertex property (absdiff). In Figure 7 the analysis of an estimator model concerning the HOXB cluster of genes according to the Lieberman-Aiden et al. Hi-C [11] experiment, which takes into account both the topological structure of the graph (edges) and the degree distribution (degree), is presented (please see Statistical Analysis S1 for the statistical analysis about the reliability of the results achieved through the MCMC simulations). In the diagnostic plots, the thick black line describes the analysed characteristics of the original graph, in particular the degree distribution (top), the edge-wise shared partner statistic (central) and the minimum geodetic distance (bottom), while the boxplot shows the statistics about the same features as stochastically simulated using the estimator. The model generated in this example is able to capture the peculiarities of the original graph, which is an important evidence that the model will be robust in describing more complex features of the model.

**Figure 8:**
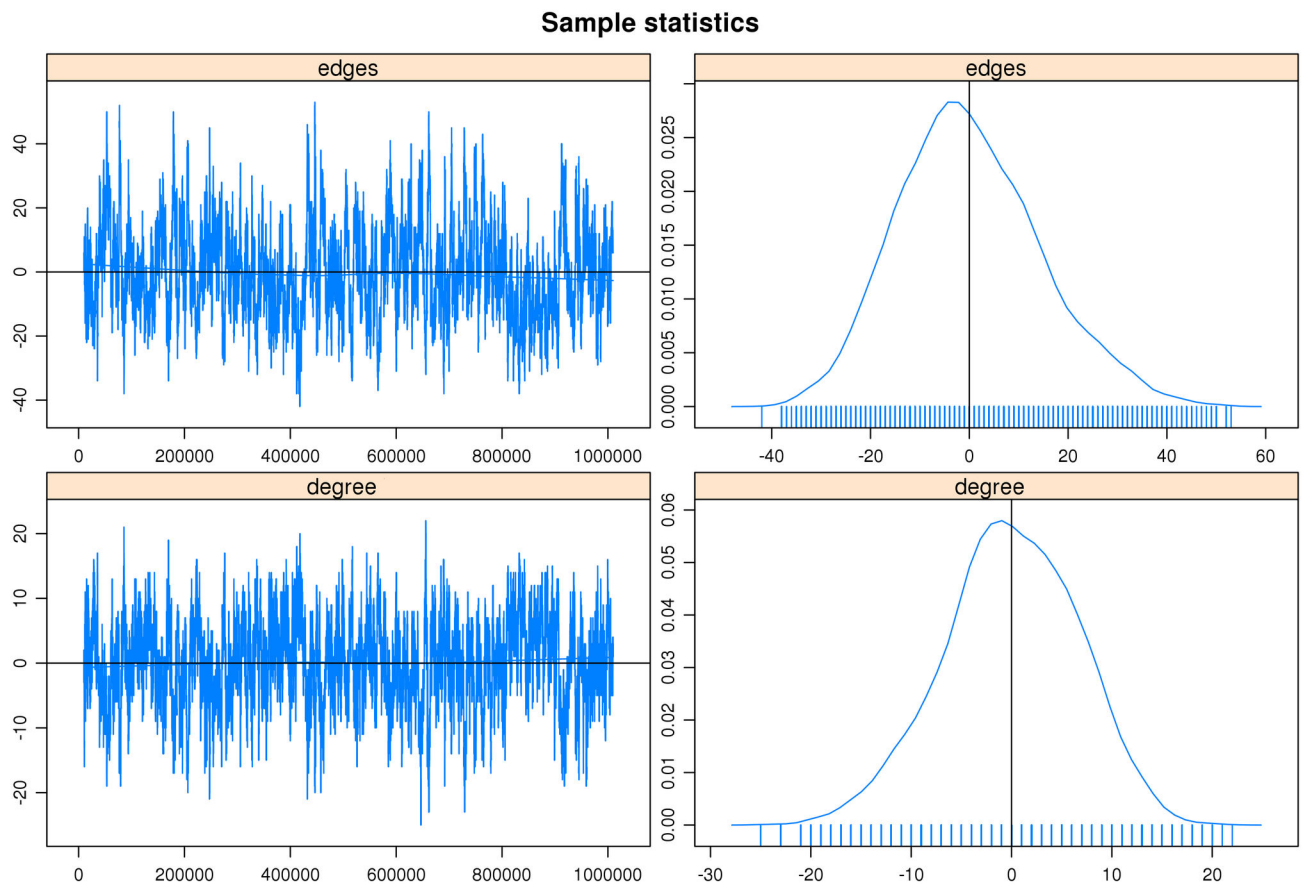
Simulation details (left) and related statistics (right) of the stochastic analysis about the two components estimator for the graph describing the HOXB cluster of genes according to the Lieberman-Aiden et al. Hi-C experiment: in the top panel the topological distribution (edges) component; in the bottom panel the degree distribution (degree) component.

In order to confirm the quality of the model estimator for the neighbourhood graph, it is possible to investigate the stochastic simulation that produced goodness-of-fit diagnostics presented above. In Figure 8 the details of the simulation (left) and the statistical analysis (right) concerning the two components of the estimator, the topological distribution (edges) and the degree distribution (degree), computed for the HOXB clusters of genes according to the Lieberman-Aiden et al. Hi-C [11] experiment presented above are shown.

Thanks to ERGMs, it is possible to understand if a particular genomic feature has a significant effect on the presence of an edge. For example, we mapped data concerning CTCF binding sites, isochores, cryptic RSSs and DNase hypersensitive sites on the achieved graph and then we used the proposed model for analysing the correlation between edges and the distribution of these genomic features. By exploring the model with a Monte Carlo simulation, we achieved in all simulations a high level of confidence (please see Statistical Analysis S1 for the statistical analysis about the reliability of the results achieved through the MCMC simulations). Noteworthy, there is a significant positive effect of DNase hypersensitive sites and CTCF binding sites (nodecov.dnase = 0.86291 ± 0.07961 and nodecov.ctcf = 0.52386 ± 0.04158) on the probability of an edge to be in the graph (see Table 3), while cryptic RSSs are less correlated with the edge distribution (nodecov.rss = 0.39780 ± 0.03176). On the contrary, isochores are negatively correlated with the presence of edges (nodecov.iso = -0.84035 ± 0.09269).

**Table 3:** Details of the stochastic simulations performed to analyse the impact of the CTCF binding site, isochores, cryptic RSSs, and DNase hypersensitive sites on the edge distribution.

Formula	Iterations	Estimate	Std. Error	p-value
edges + nodecov("dnase")	100	0.86291	0.07961	<1e-04
edges + nodecov("ctcf")	100	0.52386	0.04158	<1e-04
edges + nodecov("rss")	100	0.39780	0.03176	<1e-04
edges + nodecov("iso")	100	-0.84035	0.09269	<1e-04

As the summary table shows, after 100 iterations the statistics achieved a high degree of confidence (P-value < 0.0001). In particular, data demonstrate that DNase hypersensitive sites and CTCF binding sites have a positive influence on the presence of edges (nodecov.dnase = 0.86291 ± 0.07961 and nodecov.ctcf = 0.52386 ± 0.04158); at the same way cryptic RSSs are positively correlated with edges (nodecov.rss = 0.39780 ± 0.03176) although with less impact; on the other hand, isochores are negatively correlated with the edge distribution (nodecov.iso = -0.84035 ± 0.09269).

## Conclusions

NuChart is designed to study Hi-C data in a systems biology oriented view, with the aim of correlating the spatial distribution of genes with the mechanism of their coregulation. NuChart combines a graph based representation of Hi-C contacts (relying on a flexible normalization of mapped fragments), the analysis of expression of colocalized genes, the possibility of annotating the neighbourhood graph with multi-omics features, and the statistical analysis of the results, providing a complete tool that can be very helpful to identify novel regulatory mechanisms and can bring at the identification of novel biomarkers.

The software is freely available from ftp://fileserver.itb.cnr.it/nuchart with all the example datasets employed for the experiments and a detailed manual of the package. Future directions are in providing more embedded features to map on the graph and in the implementation of more sophisticate methods for graphs analysis and comparison. From the computational point of view, we aim at improving the quality of representations and the speed of graph computations by employing high performance on-chip architectures for the analysis, in order to produce more complete nuclear charts.

## Supporting Information

Statistical Analysis S1 **Full description of the stochastic simulations and the related statistical analyses performed for the creation of the neighbourhood graph estimators presented in the results and discussion section.**
(PDF)
