# Supplementary material for: NuChart: An R Package to Study Gene Spatial Neighbourhoods with Multi-Omics Annotations
Source: PLoS One. 2013 Sep 19;8(9):e75146. doi: 10.1371/journal.pone.0075146 (PMC3777921; doi:10.1371/journal.pone.0075146)
Supplement: Statistical Analysis S1 — Full description of the stochastic simulations and the related statistical analyses performed for the creation of the neighbourhood graph estimators presented in the results and discussion section. (PDF) [file pone.0075146.s001.pdf]

```
> model_base <- ergm(n1 ~ edges+degree(1))

Iteration 1 of at most 20:
Convergence test P-value: 2.3e-27
The log-likelihood improved by 0.5436
Iteration 2 of at most 20:
Convergence test P-value: 3.3e-19
The log-likelihood improved by 0.3567
Iteration 3 of at most 20:
Convergence test P-value: 2.9e-15
The log-likelihood improved by 0.3184
Iteration 4 of at most 20:
Convergence test P-value: 3e-42
The log-likelihood improved by 0.2939
Iteration 5 of at most 20:
Convergence test P-value: 3.4e-21
The log-likelihood improved by 0.1283
Iteration 6 of at most 20:
Convergence test P-value: 4.1e-14
The log-likelihood improved by 0.05055
Iteration 7 of at most 20:
Convergence test P-value: 9.1e-05
The log-likelihood improved by 0.01616
Iteration 8 of at most 20:
Convergence test P-value: 2.2e-01
The log-likelihood improved by 0.003225
Iteration 9 of at most 20:
Convergence test P-value: 4.4e-01
The log-likelihood improved by 0.001786
Iteration 10 of at most 20:
Convergence test P-value: 8.5e-01
Convergence detected. Stopping.
The log-likelihood improved by 0.0005741

> summary(model_base)

==========================
Summary of model fit
==========================

Formula:   n1 ~ edges + degree(1)

Iterations:  20

Monte Carlo MLE Results:
                 Estimate Std. Error MCMC % p-value
edges            -3.21882    0.20173     20  <1e-04 ***
degree1           2.63232    0.37375     20  <1e-04 ***
---
Signif. codes:  0 "***" 0.001 "**" 0.01 "*" 0.05 "." 0.1 " " 1

     Null Deviance: 7714.7  on 5565  degrees of freedom
 Residual Deviance:  955.3  on 5562  degrees of freedom

AIC: 961.3    BIC: 981.2    (Smaller is better.)
```

```
> gof(model_base)

Goodness-of-fit for degree

   obs min  mean max MC p-value
0    0   0  1.49   5       0.40
1   67  49 67.11  81       1.00
2   12   3  7.77  16       0.08
3   17   2  8.39  15       0.00
4    2   2  7.78  19       0.02
5    1   0  5.22  13       0.04
6    2   0  3.65  11       0.70
7    1   0  1.61   6       1.00
8    0   0  0.89   4       0.88
9    0   0  0.49   2       1.00
10   0   0  0.30   2       1.00
11   0   0  0.13   2       1.00
12   0   0  0.08   1       1.00
13   1   0  0.02   1       0.04
14   1   0  0.04   1       0.08
15   1   0  0.01   1       0.02
16   0   0  0.01   1       1.00
17   0   0  0.02   1       1.00
18   0   0  0.03   1       1.00
19   0   0  0.04   1       1.00
20   0   0  0.09   1       1.00
21   0   0  0.03   1       1.00
22   0   0  0.11   1       1.00
23   0   0  0.06   1       1.00
24   1   0  0.11   1       0.22
25   0   0  0.08   1       1.00
26   0   0  0.08   1       1.00
27   0   0  0.05   1       1.00
28   0   0  0.08   1       1.00
29   0   0  0.05   1       1.00
30   0   0  0.04   1       1.00
31   0   0  0.05   1       1.00
32   0   0  0.02   1       1.00
33   0   0  0.02   1       1.00
34   0   0  0.03   1       1.00
35   0   0  0.01   1       1.00
37   0   0  0.01   1       1.00

Goodness-of-fit for edgewise shared partner

     obs min  mean max MC p-value
esp0 120  80 96.95 127       0.02
esp1   0   6 18.19  39       0.00
esp2   0   0  4.40  14       0.16
esp3   0   0  1.00   7       0.90
esp4   0   0  0.25   2       1.00
esp5   0   0  0.03   1       1.00
esp6   0   0  0.01   1       1.00
esp9   0   0  0.01   1       1.00

Goodness-of-fit for minimum geodesic distance

     obs min    mean  max MC p-value
```

```
1     120  91  120.84  156       0.98
2     655 266  549.42  839       0.42
3     809 437 1020.72 1664       0.42
4    1414 469 1013.03 1436       0.04
5     837 214  591.72 1005       0.18
6     741  18  221.17  554       0.00
7     380   0   58.46  237       0.00
8      74   0   11.93  123       0.06
9      30   0    2.78   64       0.06
10      0   0    0.77   26       1.00
11      0   0    0.09    5       1.00
12      0   0    0.01    1       1.00
Inf  505 905 1974.06 3738       0.00

> mcmc.diagnostics(model_base)
Sample statistics summary:

Iterations = 10000:1009900
Thinning interval = 100
Number of chains = 1
Sample size per chain = 10000

1. Empirical mean and standard deviation for each variable,
   plus standard error of the mean:

                 Mean    SD Naive SE Time-series SE
edges         -0.1439 14.97   0.1497         1.1506
degree1        0.1558  6.67   0.0667         0.4535

2. Quantiles for each variable:

                2.5% 25% 50% 75% 97.5%
edges            -26 -11  -1  10    31
degree1          -13  -4   0   5    12


Are sample statistics significantly different from observed?
                edges   degree1  Overall (Chi^2)
diff.      -0.1439000 0.1558000               NA
test stat. -0.1250666 0.3435344        0.8063061
P-val.      0.9004709 0.7311965        0.8479581

Sample statistics cross-correlations:
                     edges    degree1 nodecov.features
edges            1.0000000 -0.8767134        0.6755583
degree1         -0.8767134  1.0000000       -0.4611390

Sample statistics auto-correlation:
Chain 1
            edges   degree1
Lag 0   1.0000000 1.0000000
Lag 100 0.9516084 0.9008448
Lag 200 0.9119373 0.8374290
Lag 300 0.8752097 0.7898382
Lag 400 0.8422160 0.7479431
Lag 500 0.8092260 0.7123876

Sample statistics burn-in diagnostic (Geweke):
Chain 1
```

```
Fraction in 1st window = 0.1
Fraction in 2nd window = 0.5

          edges          degree1
        -0.8652           0.8777

P-values (lower = worse):
          edges          degree1
      0.3869519        0.3801096
```

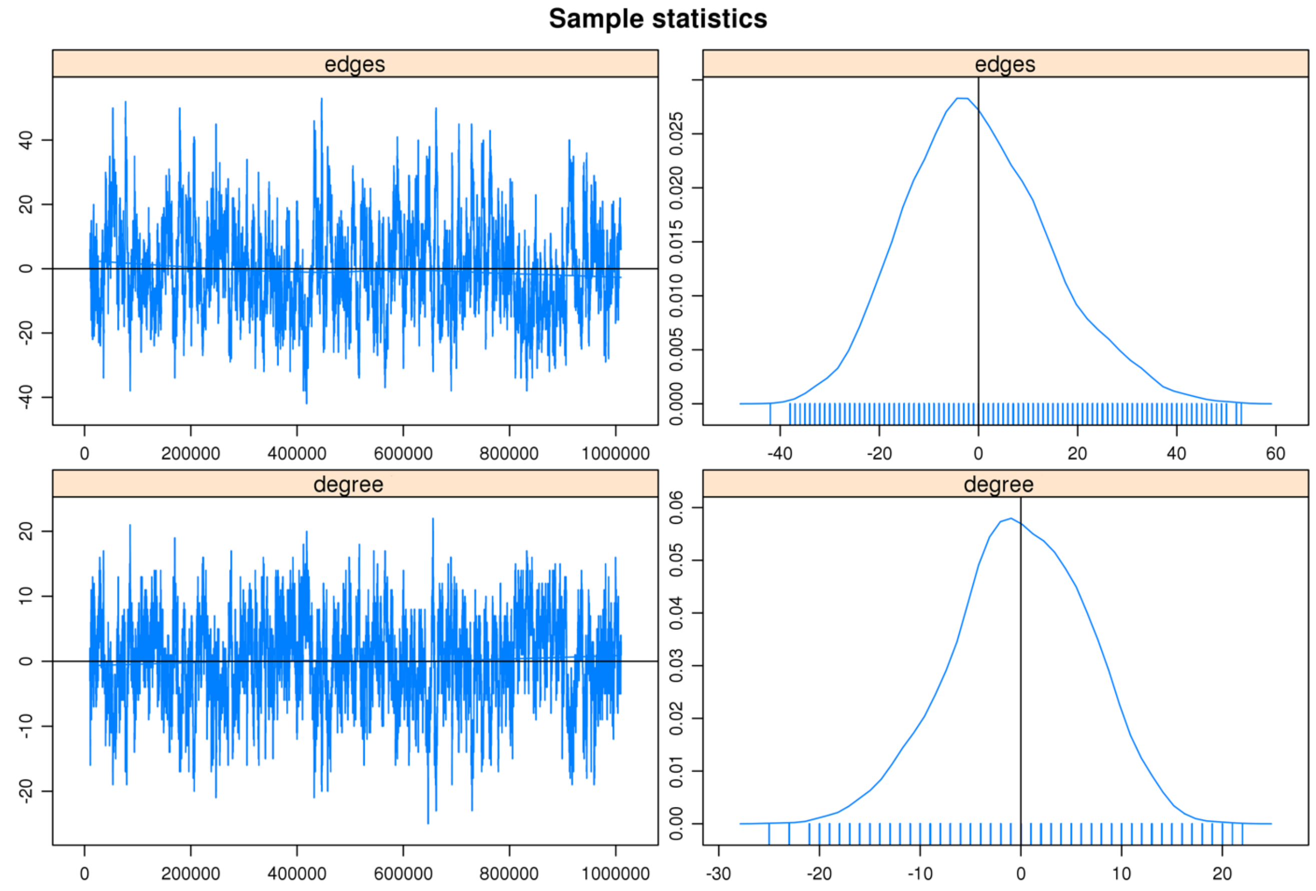

Supplementary Figure 1. Simulation details (left) and related statistics (right) of the stochastic analysis about the two components estimator for the graph describing the HOXB cluster of genes according to the Lieberman-Aiden et al. Hi-C experiment.

```
> model_ctcf <- ergm(n1 ~ edges+degree(1)+nodecov("ctcf"))

Iteration 1 of at most 20:
Convergence test P-value: 1.1e-46
The log-likelihood improved by 0.7188
Iteration 2 of at most 20:
Convergence test P-value: 3.7e-45
The log-likelihood improved by 0.4368
Iteration 3 of at most 20:
Convergence test P-value: 5.9e-25
The log-likelihood improved by 0.2327
Iteration 4 of at most 20:
Convergence test P-value: 1.3e-29
The log-likelihood improved by 0.1199
Iteration 5 of at most 20:
Convergence test P-value: 1.6e-11
The log-likelihood improved by 0.04484
Iteration 6 of at most 20:
Convergence test P-value: 2.8e-04
The log-likelihood improved by 0.01505
Iteration 7 of at most 20:
Convergence test P-value: 4.3e-04
The log-likelihood improved by 0.01176
Iteration 8 of at most 20:
Convergence test P-value: 2.6e-01
The log-likelihood improved by 0.0027
Iteration 9 of at most 20:
Convergence test P-value: 3e-01
The log-likelihood improved by 0.002686
Iteration 10 of at most 20:
Convergence test P-value: 7.6e-01
Convergence detected. Stopping.
The log-likelihood improved by 0.0007933

> summary(model_ctcf)

==========================
Summary of model fit
==========================

Formula:   n1 ~ edges + degree(1) + nodecov("ctcf")

Iterations:  20

Monte Carlo MLE Results:
             Estimate Std. Error MCMC % p-value
edges        -3.22486    0.20126     17  <1e-04 ***
degree1       2.62902    0.36735     16  <1e-04 ***
nodecov.ctcf  0.52386    0.04158      6  <1e-04 ***
---
Signif. codes:  0 "***" 0.001 "**" 0.01 "*" 0.05 "." 0.1 " " 1


     Null Deviance: 7714.7  on 5565  degrees of freedom
 Residual Deviance:  954.5  on 5562  degrees of freedom

AIC: 960.5    BIC: 980.4    (Smaller is better.)
```

```
> gof(model_ctcf)

Goodness-of-fit for degree

   obs min  mean max MC p-value
0    0   0  1.39   5       0.44
1   67  55 68.07  87       0.98
2   12   2  7.85  15       0.18
3   17   2  8.71  16       0.00
4    2   1  7.65  16       0.04
5    1   0  4.91  11       0.10
6    2   0  3.05   9       0.92
7    1   0  1.55   7       1.00
8    0   0  0.84   5       0.80
9    0   0  0.41   3       1.00
10   0   0  0.26   2       1.00
11   0   0  0.12   2       1.00
12   0   0  0.10   1       1.00
13   1   0  0.02   1       0.04
14   1   0  0.06   1       0.12
15   1   0  0.02   1       0.04
16   0   0  0.01   1       1.00
17   0   0  0.07   1       1.00
18   0   0  0.03   1       1.00
19   0   0  0.02   1       1.00
20   0   0  0.03   1       1.00
21   0   0  0.09   1       1.00
22   0   0  0.08   1       1.00
23   0   0  0.07   1       1.00
24   1   0  0.08   1       0.16
25   0   0  0.11   1       1.00
26   0   0  0.09   1       1.00
27   0   0  0.05   1       1.00
28   0   0  0.07   1       1.00
29   0   0  0.09   1       1.00
30   0   0  0.04   1       1.00
31   0   0  0.02   1       1.00
32   0   0  0.02   1       1.00
33   0   0  0.01   1       1.00
38   0   0  0.01   1       1.00

Goodness-of-fit for edgewise shared partner

     obs min  mean max MC p-value
esp0 120  76 97.88 120       0.02
esp1   0   0 15.54  36       0.02
esp2   0   0  3.29  13       0.40
esp3   0   0  0.83   5       1.00
esp4   0   0  0.11   3       1.00
esp5   0   0  0.03   1       1.00
```

```
Goodness-of-fit for minimum geodesic distance

     obs min    mean  max MC p-value
1    120  86  117.68  144       0.82
2    655 221  519.89  924       0.20
```

```
3    809 301  954.78 1496       0.60
4   1414 238  970.08 1471       0.02
5    837  78  582.70  994       0.16
6    741  30  231.17  616       0.00
7    380   0   72.54  281       0.00
8     74   0   20.04  256       0.18
9     30   0    6.80  203       0.12
10     0   0    2.63  130       1.00
11     0   0    0.76   52       1.00
12     0   0    0.16   16       1.00
Inf  505 511 2085.77 4194       0.00

> mcmc.diagnostics(model_ctcf)
Sample statistics summary:

Iterations = 10000:1009900
Thinning interval = 100
Number of chains = 1
Sample size per chain = 10000

1. Empirical mean and standard deviation for each variable,
   plus standard error of the mean:

                Mean     SD Naive SE Time-series SE
edges        -0.8918 13.957  0.13957         0.9713
degree1       0.2751  6.575  0.06575         0.4017
nodecov.ctcf -2.4106 32.056  0.32056         2.4000

2. Quantiles for each variable:

             2.5% 25% 50% 75% 97.5%
edges         -26 -11  -2   8 29.00
degree1       -13  -4   1   5 12.02
nodecov.ctcf  -66 -24  -2  20 58.00


Are sample statistics significantly different from observed?
                edges   degree1 nodecov.ctcf Overall (Chi^2)
diff.      -0.8918000 0.2751000   -1.4106000              NA
test stat. -0.9181081 0.6848680   -1.0044315       1.1555172
P-val.      0.3585622 0.4934272    0.3151707       0.7636917

Sample statistics cross-correlations:
                  edges    degree1 nodecov.ctcf
edges         1.0000000 -0.8753849    0.6168059
degree1      -0.8753849  1.0000000   -0.4054477
nodecov.ctcf  0.6168059 -0.4054477    1.0000000

Sample statistics auto-correlation:
Chain 1
            edges   degree1 nodecov.ctcf
Lag 0   1.0000000 1.0000000    1.0000000
Lag 100 0.9449862 0.8970551    0.9613638
Lag 200 0.8985582 0.8262543    0.9260649
Lag 300 0.8582403 0.7722118    0.8939116
Lag 400 0.8207605 0.7273405    0.8629792
Lag 500 0.7861635 0.6889831    0.8338639

Sample statistics burn-in diagnostic (Geweke):
Chain 1
```

```
Fraction in 1st window = 0.1
Fraction in 2nd window = 0.5

       edges      degree1 nodecov.ctcf
     -0.3159      -0.1597      -1.0137

P-values (lower = worse):
       edges      degree1 nodecov.ctcf
   0.7520804    0.8731382    0.3107134
```

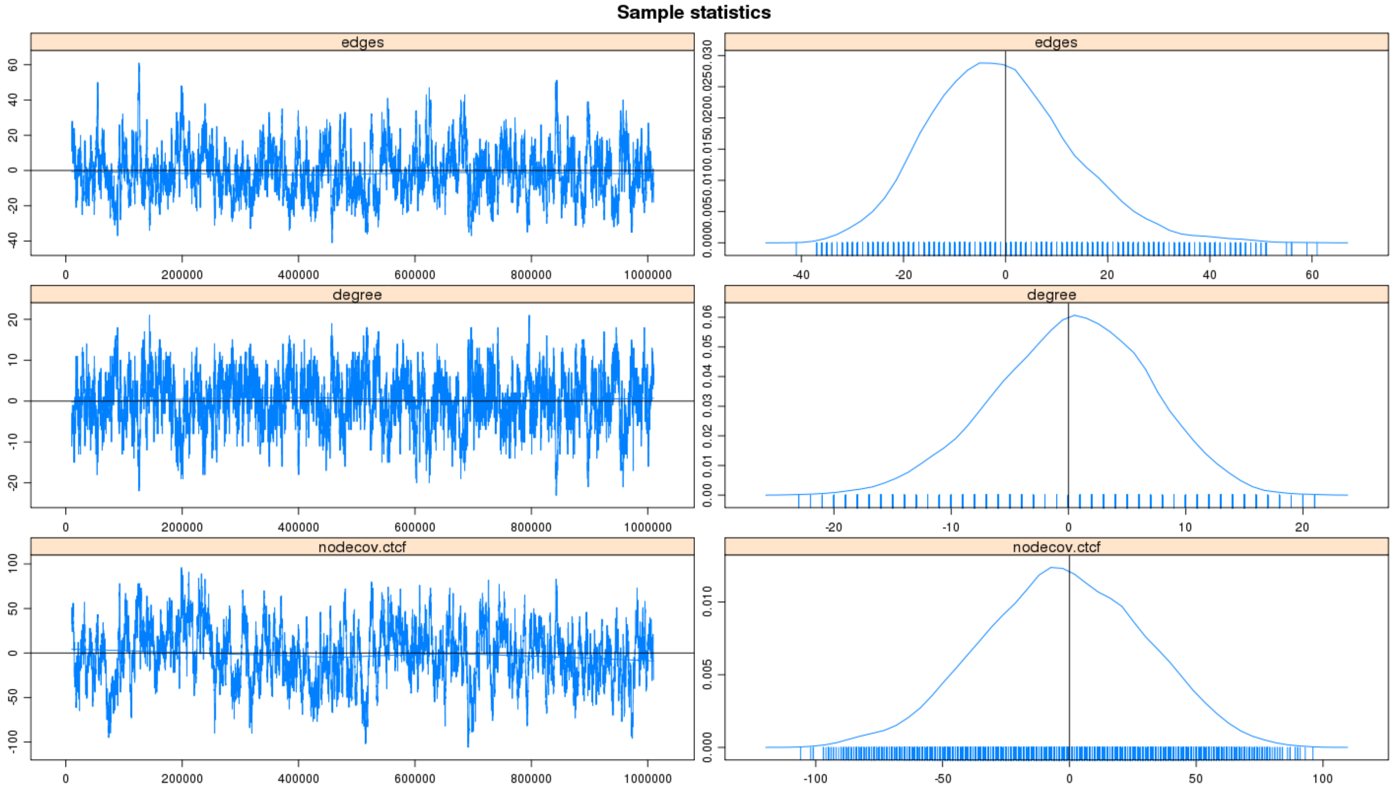

Supplementary Figure 2. Simulation details (left) and related statistics (right) of the stochastic analysis about the three components estimator (edges, degree, and nodecov.ctcf) for the graph describing the HOXB cluster of genes according to the Lieberman-Aiden et al. Hi-C experiment.

> model_rss <- ergm(n1 ~ edges+degree(1)+nodecov("rss"))

```
Iteration 1 of at most 20:
Convergence test P-value: 4.2e-213
The log-likelihood improved by 1.639
Iteration 2 of at most 20:
Convergence test P-value: 2.7e-27
The log-likelihood improved by 0.5103
Iteration 3 of at most 20:
Convergence test P-value: 1.1e-34
The log-likelihood improved by 0.5345
Iteration 4 of at most 20:
Convergence test P-value: 4.8e-33
The log-likelihood improved by 0.3403
Iteration 5 of at most 20:
Convergence test P-value: 1.1e-40
The log-likelihood improved by 0.2575
Iteration 6 of at most 20:
Convergence test P-value: 7.9e-28
The log-likelihood improved by 0.1187
Iteration 7 of at most 20:
Convergence test P-value: 8.4e-14
The log-likelihood improved by 0.04614
Iteration 8 of at most 20:
Convergence test P-value: 7.3e-02
The log-likelihood improved by 0.004191
Iteration 9 of at most 20:
Convergence test P-value: 4.4e-01
The log-likelihood improved by 0.001731
Iteration 10 of at most 20:
Convergence test P-value: 3.4e-01
The log-likelihood improved by 0.00267
Iteration 11 of at most 20:
Convergence test P-value: 3.7e-01
The log-likelihood improved by 0.001355
Iteration 12 of at most 20:
Convergence test P-value: 8.3e-03
The log-likelihood improved by 0.004198
Iteration 13 of at most 20:
Convergence test P-value: 2.9e-01
The log-likelihood improved by 0.0019
Iteration 14 of at most 20:
Convergence test P-value: 3.4e-01
The log-likelihood improved by 0.001825
Iteration 15 of at most 20:
Convergence test P-value: 9.9e-02
The log-likelihood improved by 0.004545
Iteration 16 of at most 20:
Convergence test P-value: 9.7e-01
```

Convergence detected. Stopping.
The log-likelihood improved by 0.0001182

```
> summary(model_rss)

==========================
Summary of model fit
==========================
```

```
Formula:   n1 ~ edges + degree(1) + nodecov("rss")

Iterations:  20

Monte Carlo MLE Results:
            Estimate Std. Error MCMC % p-value
edges       -3.36306    0.22717     28  <1e-04 ***
degree1      2.60012    0.38793     25  <1e-04 ***
nodecov.rss  0.39780    0.03176     12  <1e-04 ***
---
Signif. codes:  0 ″***″ 0.001 “**” 0.01 “*” 0.05 “.” 0.1 “ ” 1

     Null Deviance: 7714.7  on 5565  degrees of freedom
 Residual Deviance:  954.1  on 5562  degrees of freedom

AIC: 960.1    BIC: 980    (Smaller is better.)

> gof(model_rss)

Goodness-of-fit for degree

   obs min  mean max MC p-value
0    0   0  1.43   5       0.52
1   67  49 67.01  78       0.92
2   12   3  8.37  14       0.38
3   17   3  8.94  19       0.06
4    2   2  7.77  15       0.04
5    1   1  4.83  12       0.08
6    2   0  2.78   7       0.92
7    1   0  1.80   6       0.96
8    0   0  0.93   4       0.82
9    0   0  0.50   2       1.00
10   0   0  0.25   2       1.00
11   0   0  0.20   2       1.00
12   0   0  0.07   1       1.00
13   1   0  0.06   1       0.12
14   1   0  0.03   1       0.06
15   1   0  0.01   1       0.02
16   0   0  0.06   1       1.00
17   0   0  0.02   1       1.00
18   0   0  0.06   1       1.00
19   0   0  0.05   1       1.00
20   0   0  0.07   1       1.00
21   0   0  0.04   1       1.00
22   0   0  0.10   1       1.00
23   0   0  0.14   1       1.00
24   1   0  0.10   1       0.20
25   0   0  0.07   1       1.00
26   0   0  0.09   1       1.00
27   0   0  0.05   1       1.00
28   0   0  0.03   1       1.00
29   0   0  0.04   1       1.00
30   0   0  0.03   1       1.00
31   0   0  0.03   1       1.00
32   0   0  0.01   1       1.00
34   0   0  0.01   1       1.00
38   0   0  0.01   1       1.00
39   0   0  0.01   1       1.00
```

```
Goodness-of-fit for edgewise shared partner

     obs min  mean max MC p-value
esp0 120  81 97.59 130       0.06
esp1   0   0 16.67  40       0.02
esp2   0   0  3.74  12       0.22
esp3   0   0  0.78   4       1.00
esp4   0   0  0.18   3       1.00
esp5   0   0  0.03   1       1.00
esp6   0   0  0.01   1       1.00

Goodness-of-fit for minimum geodesic distance

     obs min    mean  max MC p-value
1    120  97  119.00  160       0.90
2    655 305  512.67  941       0.28
3    809 484  951.40 1558       0.66
4   1414 439  972.45 1603       0.10
5    837 234  594.14  948       0.18
6    741  49  237.53  532       0.00
7    380   2   70.43  270       0.00
8     74   0   16.48  114       0.08
9     30   0    3.41   65       0.04
10     0   0    0.65   32       1.00
11     0   0    0.21   15       1.00
Inf  505 412 2086.63 3609       0.02

> mcmc.diagnostics(model_rss)
Sample statistics summary:

Iterations = 10000:1009900
Thinning interval = 100
Number of chains = 1
Sample size per chain = 10000

1. Empirical mean and standard deviation for each variable,
   plus standard error of the mean:

               Mean     SD Naive SE Time-series SE
edges        0.0505 14.077  0.14077         0.9633
degree1     -0.1124  6.372  0.06372         0.3876
nodecov.rss  0.0745 47.443  0.47443         3.5640

2. Quantiles for each variable:

            2.5% 25% 50% 75% 97.5%
edges        -25 -10  -1   9    31
degree1      -13  -4   0   4    12
nodecov.rss  -90 -32  -1  33    93


Are sample statistics significantly different from observed?
               edges    degree1 nodecov.rss Overall (Chi^2)
diff.      0.0505000 -0.1124000  0.07450000              NA
test stat. 0.0524262 -0.2899927  0.02090356       0.2673271
P-val.     0.9581891  0.7718218  0.98332259       0.9660515

Sample statistics cross-correlations:
                 edges    degree1 nodecov.rss
edges        1.0000000 -0.8706452   0.7568610
```

```
degree1      -0.8706452  1.0000000  -0.5582727
nodecov.rss   0.7568610 -0.5582727   1.0000000

Sample statistics auto-correlation:
Chain 1
               edges   degree1 nodecov.rss
Lag 0    1.0000000 1.0000000   1.0000000
Lag 100 0.9459533 0.8933994   0.9634909
Lag 200 0.8991287 0.8238714   0.9300327
Lag 300 0.8585887 0.7727913   0.8985329
Lag 400 0.8181824 0.7267730   0.8671116
Lag 500 0.7807026 0.6866192   0.8378673

Sample statistics burn-in diagnostic (Geweke):
Chain 1

Fraction in 1st window = 0.1
Fraction in 2nd window = 0.5

      edges      degree1 nodecov.rss
     -1.309        1.319      -1.213

P-values (lower = worse):
      edges      degree1 nodecov.rss
  0.1905293    0.1870648    0.2249617
```

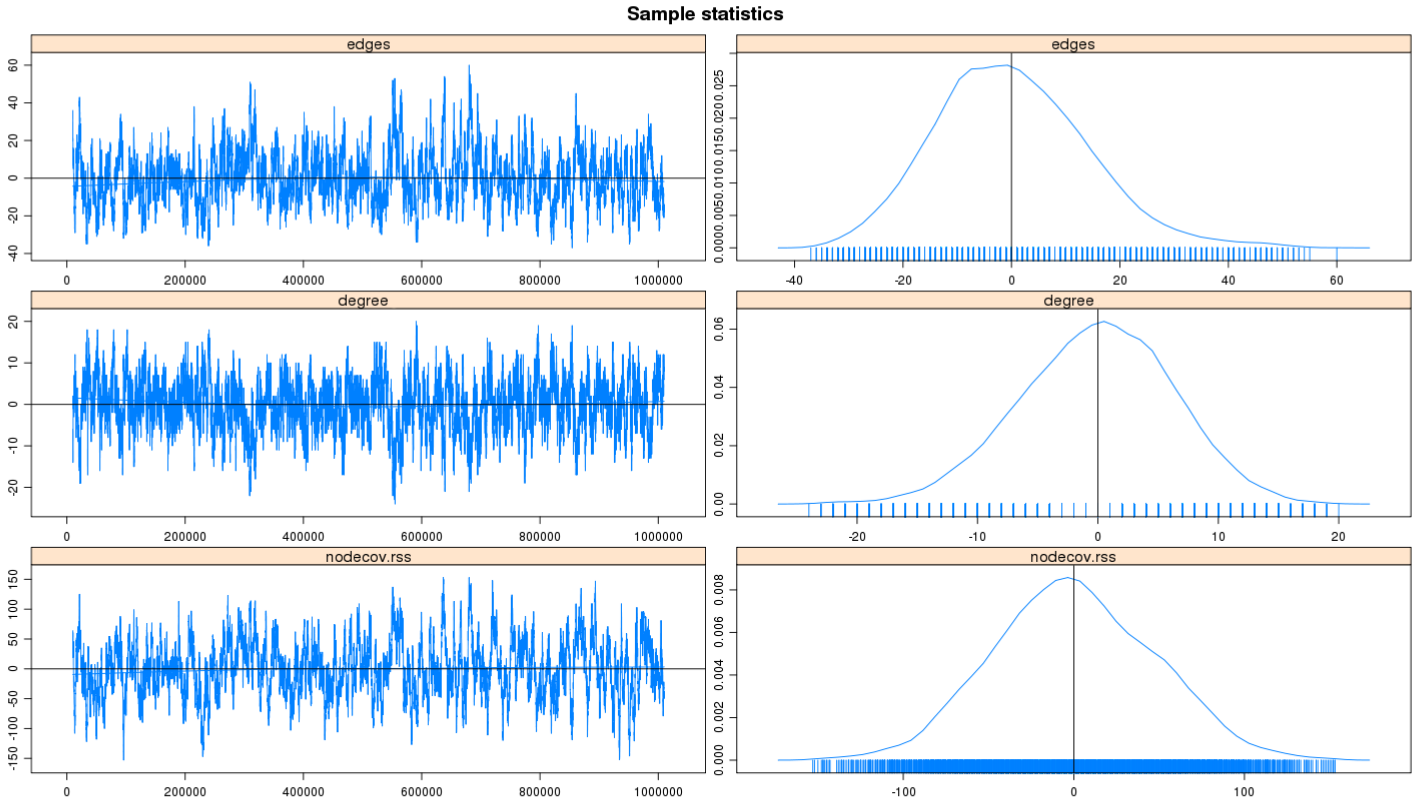

Supplementary Figure 3. Simulation details (left) and related statistics (right) of the stochastic analysis about the three components estimator (edges, degree, and nodecov.rss) for the graph describing the HOXB cluster of genes according to the Lieberman-Aiden et al. Hi-C experiment.

```
> model_iso <- ergm(n1 ~ edges+degree(1)+nodecov("iso"))

Iteration 1 of at most 20:
Convergence test P-value: 0e+00
The log-likelihood improved by 1.494
Iteration 2 of at most 20:
Convergence test P-value: 4.2e-97
The log-likelihood improved by 0.2085
Iteration 3 of at most 20:
Convergence test P-value: 2.4e-16
The log-likelihood improved by 0.04267
Iteration 4 of at most 20:
Convergence test P-value: 1.6e-04
The log-likelihood improved by 0.01003
Iteration 5 of at most 20:
Convergence test P-value: 9.6e-04
The log-likelihood improved by 0.007459
Iteration 6 of at most 20:
Convergence test P-value: 8e-02
The log-likelihood improved by 0.004762
Iteration 7 of at most 20:
Convergence test P-value: 5.2e-01
Convergence detected. Stopping.
The log-likelihood improved by 0.002215

> summary(model_iso)

==========================
Summary of model fit
==========================

Formula:   n1 ~ edges + degree(1) + nodecov("iso")

Iterations:  20

Monte Carlo MLE Results:
             Estimate Std. Error MCMC %  p-value
edges       -3.307232   0.438861     83  < 1e-04 ***
degree1      2.474114   0.693693     79 0.000365 ***
nodecov.iso -0.840357   0.092697     62  < 1e-04 ***
---
Signif. codes:  0 "***" 0.001 "**" 0.01 "*" 0.05 "." 0.1 " " 1

     Null Deviance: 7714.7  on 5565  degrees of freedom
 Residual Deviance:  918.2  on 5562  degrees of freedom

AIC: 924.2    BIC: 944    (Smaller is better.)


> gof(model_iso)

Goodness-of-fit for degree

   obs min  mean max MC p-value
0    0   0  1.75   6       0.34
1   67  54 68.76  83       0.82
2   12   1  8.54  15       0.30
3   17   2  9.00  18       0.04
4    2   2  6.96  16       0.04
```

5	1	0	4.54	12	0.16
6	2	0	2.51	7	1.00
7	1	0	1.15	5	1.00
8	0	0	0.65	3	0.98
9	0	0	0.51	3	1.00
10	0	0	0.32	2	1.00
11	0	0	0.15	2	1.00
12	0	0	0.07	1	1.00
13	1	0	0.05	2	0.08
14	1	0	0.02	1	0.04
15	1	0	0.02	1	0.04
20	0	0	0.01	1	1.00
22	0	0	0.01	1	1.00
24	1	0	0.01	1	0.02
25	0	0	0.02	1	1.00
26	0	0	0.05	1	1.00
27	0	0	0.08	1	1.00
28	0	0	0.03	1	1.00
29	0	0	0.10	1	1.00
30	0	0	0.06	1	1.00
31	0	0	0.06	1	1.00
32	0	0	0.08	1	1.00
33	0	0	0.05	1	1.00
34	0	0	0.12	1	1.00
35	0	0	0.10	1	1.00
36	0	0	0.05	1	1.00
37	0	0	0.03	1	1.00
38	0	0	0.04	1	1.00
39	0	0	0.03	1	1.00
40	0	0	0.03	1	1.00
41	0	0	0.01	1	1.00
42	0	0	0.01	1	1.00
43	0	0	0.01	1	1.00
49	0	0	0.01	1	1.00

Goodness-of-fit for edgewise shared partner

	obs	min	mean	max	MC p-value
esp0	120	73	92.59	116	0.00
esp1	0	6	18.19	35	0.00
esp2	0	0	5.19	14	0.08
esp3	0	0	1.36	8	0.68
esp4	0	0	0.31	3	1.00
esp5	0	0	0.08	2	1.00
esp6	0	0	0.06	1	1.00

Goodness-of-fit for minimum geodesic distance

	obs	min	mean	max	MC p-value
1	120	91	117.78	149	0.78
2	655	404	709.56	1267	0.80
3	809	608	1151.63	1740	0.12
4	1414	431	959.08	1411	0.00
5	837	166	463.41	997	0.04
6	741	9	149.05	417	0.00
7	380	0	34.75	207	0.00
8	74	0	5.29	57	0.00
9	30	0	0.49	9	0.00
10	0	0	0.04	3	1.00
Inf	505	808	1973.92	3714	0.00

```
> mcmc.diagnostics(model_iso)
Sample statistics summary:

Iterations = 10000:1009900
Thinning interval = 100
Number of chains = 1
Sample size per chain = 10000

1. Empirical mean and standard deviation for each variable,
   plus standard error of the mean:

                Mean      SD Naive SE Time-series SE
edges        -0.5141  13.547  0.13547         0.8256
degree1       0.0550   6.715  0.06715         0.3728
nodecov.iso  -8.2234  24.476  2.24476         9.5127

2. Quantiles for each variable:

               2.5%  25% 50% 75% 97.5%
edges        -24.00  -10  -2   8    28
degree1      -13.02   -4   0   5    13
nodecov.iso  -45.02  -18 -38  12    19


Are sample statistics significantly different from observed?
                edges   degree1 nodecov.iso Overall (Chi^2)
diff.      -0.5141000 0.0550000  -2.2234000              NA
test stat. -0.6227272 0.1475413  -0.4464095       1.2645635
P-val.      0.5334638 0.8827047   0.1480624       0.5193436

Sample statistics cross-correlations:
                 edges    degree1 nodecov.iso
edges        1.0000000 -0.8637495   0.5834390
degree1     -0.8637495  1.0000000  -0.3559374
nodecov.iso  0.5834390 -0.3559374   1.0000000

Sample statistics auto-correlation:
Chain 1
            edges   degree1 nodecov.iso
Lag 0   1.0000000 1.0000000   1.0000000
Lag 100 0.9418846 0.8945609   0.9724958
Lag 200 0.8910281 0.8251952   0.9471588
Lag 300 0.8448309 0.7672479   0.9230766
Lag 400 0.8019794 0.7181222   0.9000278
Lag 500 0.7615925 0.6739926   0.8768215

Sample statistics burn-in diagnostic (Geweke):
Chain 1

Fraction in 1st window = 0.1
Fraction in 2nd window = 0.5

      edges     degree1 nodecov.iso
    0.61736     0.06172     2.15205

P-values (lower = worse):
      edges     degree1 nodecov.iso
 0.53699653  0.95078271  0.31393487
```

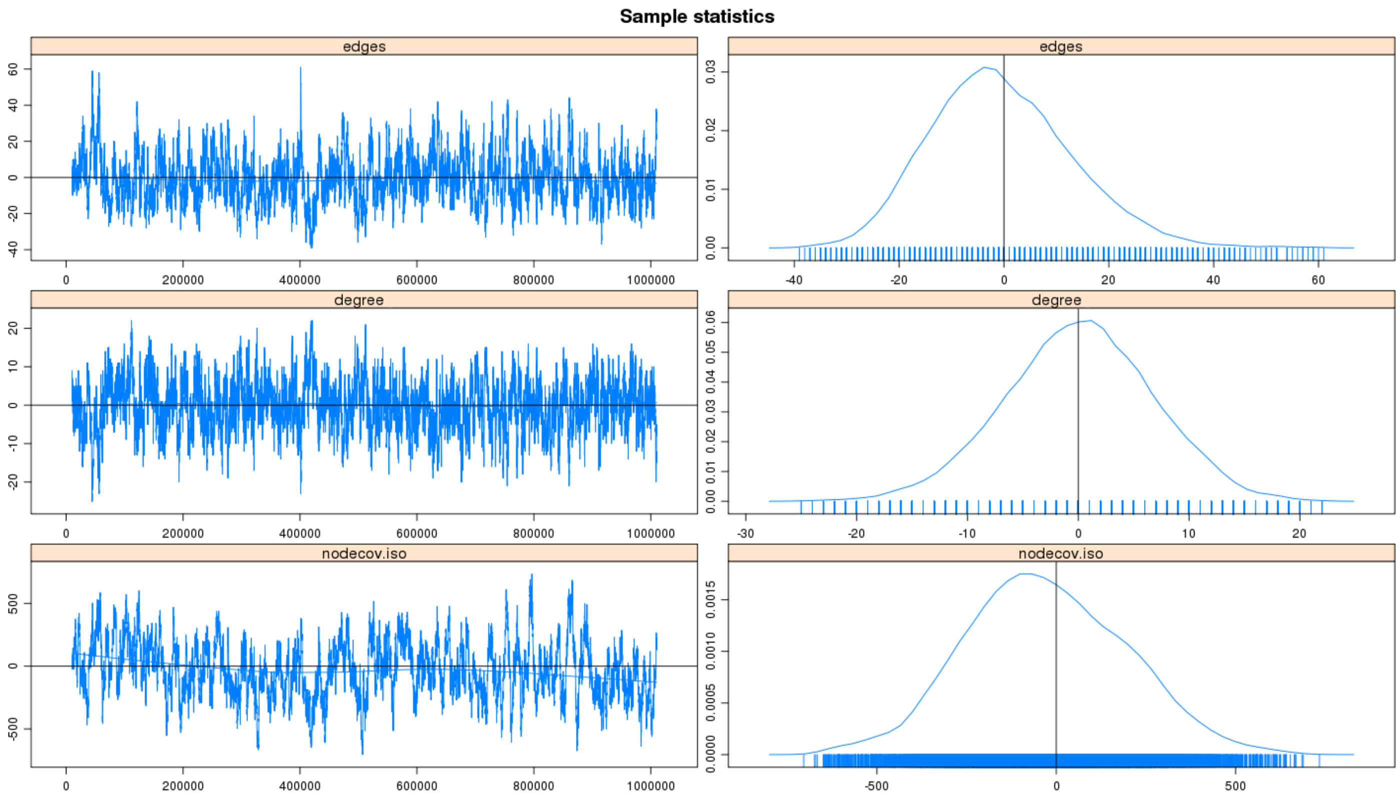

Supplementary Figure 4. Simulation details (left) and related statistics (right) of the stochastic analysis about the three components estimator (edges, degree, and nodecov.iso) for the graph describing the HOXB cluster of genes according to the Lieberman-Aiden et al. Hi-C experiment.

> model_dnase <- ergm(n1 ~ edges+degree(1)+nodecov("dnase"))

```
Iteration 1 of at most 20:
Convergence test P-value: 0e+00
The log-likelihood improved by 5.794
Iteration 2 of at most 20:
Convergence test P-value: 1.1e-60
The log-likelihood improved by 1.245
Iteration 3 of at most 20:
Convergence test P-value: 6.6e-90
The log-likelihood improved by 0.6984
Iteration 4 of at most 20:
Convergence test P-value: 3.5e-89
The log-likelihood improved by 0.4642
Iteration 5 of at most 20:
Convergence test P-value: 7.7e-37
The log-likelihood improved by 0.1958
Iteration 6 of at most 20:
Convergence test P-value: 1.3e-22
The log-likelihood improved by 0.08744
Iteration 7 of at most 20:
Convergence test P-value: 1.3e-05
The log-likelihood improved by 0.01626
Iteration 8 of at most 20:
Convergence test P-value: 3.9e-03
The log-likelihood improved by 0.005713
Iteration 9 of at most 20:
Convergence test P-value: 2.4e-01
The log-likelihood improved by 0.001532
Iteration 10 of at most 20:
Convergence test P-value: 5.1e-01
Convergence detected. Stopping.
The log-likelihood improved by 0.0009326

> summary(model_dnase)

==========================
Summary of model fit
==========================

Formula:   n1 ~ edges + degree(1) + nodecov("dnase")

Iterations:  20

Monte Carlo MLE Results:
              Estimate Std. Error MCMC % p-value
edges         -3.38574    0.22362     12  <1e-04 ***
degree1        2.59670    0.35767     12  <1e-04 ***
nodecov.dnase  0.86291    0.07961      6  <1e-04 ***
---
Signif. codes:  0 ″***″ 0.001 “**” 0.01 “*” 0.05 “.” 0.1 “ “ 1

     Null Deviance: 7714.7  on 5565  degrees of freedom
 Residual Deviance:  974.6  on 5562  degrees of freedom

AIC: 980.6    BIC: 1000    (Smaller is better.)

> gof(model_dnase)
```

```
Goodness-of-fit for degree

   obs min  mean max MC p-value
0    0   0  1.52   4       0.32
1   67  46 66.46  80       1.00
2   12   1  7.14  17       0.08
3   17   2  8.51  17       0.04
4    2   0  6.69  14       0.16
5    1   1  5.46  13       0.04
6    2   0  3.45  10       0.74
7    1   0  2.14   6       0.78
8    0   0  1.43   5       0.38
9    0   0  0.91   4       0.78
10   0   0  0.65   3       1.00
11   0   0  0.51   2       1.00
12   0   0  0.27   2       1.00
13   1   0  0.19   2       0.36
14   1   0  0.16   1       0.32
15   1   0  0.13   1       0.26
16   0   0  0.10   1       1.00
17   0   0  0.08   2       1.00
18   0   0  0.05   1       1.00
19   0   0  0.06   1       1.00
20   0   0  0.06   1       1.00
22   0   0  0.01   1       1.00
23   0   0  0.01   1       1.00
24   1   0  0.01   1       0.02

Goodness-of-fit for edgewise shared partner

     obs min   mean max MC p-value
esp0 120  84 101.00 125       0.12
esp1   0   3  16.82  34       0.00
esp2   0   0   3.64  15       0.26
esp3   0   0   0.66   5       1.00
esp4   0   0   0.13   2       1.00
esp5   0   0   0.02   1       1.00

Goodness-of-fit for minimum geodesic distance

     obs min    mean  max MC p-value
1    120  96  122.27  173       0.98
2    655 245  417.37  736       0.02
3    809 449  875.90 1541       0.84
4   1414 537 1036.78 1626       0.12
5    837 307  710.92 1043       0.44
6    741  55  304.11  704       0.00
7    380   0   94.74  510       0.04
8     74   0   26.29  225       0.20
9     30   0    7.49  133       0.20
10     0   0    2.09   82       1.00
11     0   0    0.35   22       1.00
12     0   0    0.06    6       1.00
Inf  505 513 1966.63 3582       0.00

> mcmc.diagnostics(model_dnase)
Sample statistics summary:

Iterations = 10000:1009900
Thinning interval = 100
```

```
Number of chains = 1
Sample size per chain = 10000

1. Empirical mean and standard deviation for each variable,
   plus standard error of the mean:

                 Mean    SD Naive SE Time-series SE
edges          1.0849 13.23   0.1323         0.7842
degree1       -0.4358  5.92   0.0592         0.3313
nodecov.dnase  1.0124 21.30   0.2130         1.4731

2. Quantiles for each variable:

              2.5% 25% 50% 75% 97.5%
edges          -23  -8   1   9    29
degree1        -13  -4   0   4    10
nodecov.dnase  -43 -13   1  16    42


Are sample statistics significantly different from observed?
               edges    degree1 nodecov.dnase Overall (Chi^2)
diff.      1.0849000 -0.4358000     1.0124000              NA
test stat. 1.3833705 -1.3154983     0.6872654       1.3255196
P-val.     0.1665513  0.1883425     0.4919155       0.5076497

Sample statistics cross-correlations:
                   edges    degree1 nodecov.dnase
edges          1.0000000 -0.8322978     0.7842621
degree1       -0.8322978  1.0000000    -0.5092799
nodecov.dnase  0.7842621 -0.5092799     1.0000000

Sample statistics auto-correlation:
Chain 1
            edges   degree1 nodecov.dnase
Lag 0   1.0000000 1.0000000     1.0000000
Lag 100 0.9391258 0.8775555     0.9543859
Lag 200 0.8877318 0.7973573     0.9135582
Lag 300 0.8400531 0.7350269     0.8765605
Lag 400 0.7958272 0.6856351     0.8415857
Lag 500 0.7545763 0.6428520     0.8070126

Sample statistics burn-in diagnostic (Geweke):
Chain 1

Fraction in 1st window = 0.1
Fraction in 2nd window = 0.5

        edges       degree1 nodecov.dnase
       0.7512       -0.7546        0.4739

P-values (lower = worse):
        edges       degree1 nodecov.dnase
    0.4525065     0.4504791     0.6355580
```

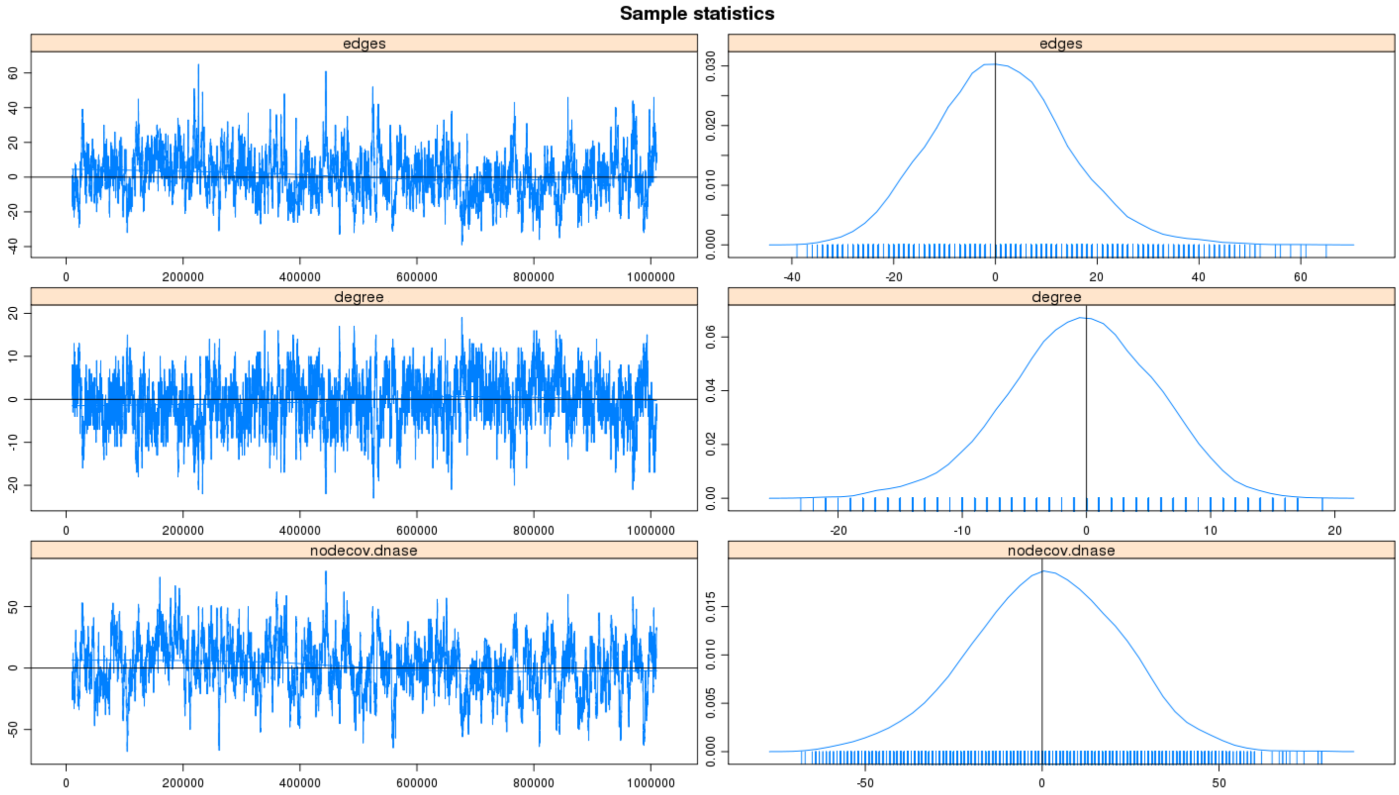

Supplementary Figure 5. Simulation details (left) and related statistics (right) of the stochastic analysis about the three components estimator (edges, degree, and nodecov.dnase) for the graph describing the HOXB cluster of genes according to the Lieberman-Aiden et al. Hi-C experiment.
